# DNA methylation regulates RNA m^6^A modification through transcription factor SP1 during the development of porcine somatic cell nuclear transfer embryos

**DOI:** 10.1111/cpr.13581

**Published:** 2023-12-14

**Authors:** Meng Zhang, Yanhui Zhai, Xinglan An, Qi Li, Daoyu Zhang, Yongfeng Zhou, Sheng Zhang, Xiangpeng Dai, Ziyi Li

**Affiliations:** ^1^ Key Laboratory of Organ Regeneration and Transplantation of Ministry of Education The First Hospital of Jilin University Changchun Jilin China

## Abstract

Epigenetic modifications play critical roles during somatic cell nuclear transfer (SCNT) embryo development. Whether RNA N6‐methyladenosine (m^6^A) affects the developmental competency of SCNT embryos remains unclear. Here, we showed that porcine bone marrow mesenchymal stem cells (pBMSCs) presented higher RNA m^6^A levels than those of porcine embryonic fibroblasts (pEFs). SCNT embryos derived from pBMSCs had higher RNA m^6^A levels, cleavage, and blastocyst rates than those from pEFs. Compared with pEFs, the promoter region of METTL14 presented a hypomethylation status in pBMSCs. Mechanistically, DNA methylation regulated METTL14 expression by affecting the accessibility of transcription factor SP1 binding, highlighting the role of the DNA methylation/SP1/METTL14 pathway in donor cells. Inhibiting the DNA methylation level in donor cells increased the RNA m^6^A level and improved the development efficiency of SCNT embryos. Overexpression of METTL14 significantly increased the RNA m^6^A level in donor cells and the development efficiency of SCNT embryos, whereas knockdown of METTL14 suggested the opposite result. Moreover, we revealed that RNA m^6^A‐regulated *TOP2B* mRNA stability, translation level, and DNA damage during SCNT embryo development. Collectively, our results highlight the crosstalk between RNA m^6^A and DNA methylation, and the crucial role of RNA m^6^A during nuclear reprogramming in SCNT embryo development.

## Graphical abstract

Crosstalk regulation between DNA methylation and RNA N6‐methyladenosine in donor cell during nuclear reprogramming in somatic cell nuclear transfer embryos.

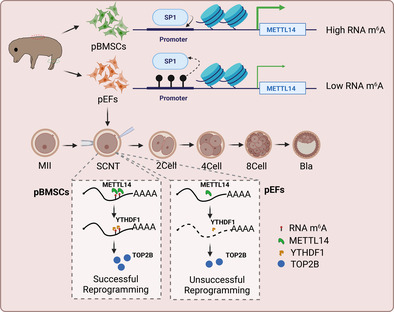

## 1 INTRODUCTION

Somatic cell nuclear transfer (SCNT) is a technology that transfers differentiated somatic cells to enucleated oocytes and then enables embryos to develop into individuals.^1^ In recent years, SCNT technology has been widely developed and applied due to our comprehensive understanding of epigenetics, such as DNA methylation, histone modifications, and non‐coding RNAs.^2^, ^3^, ^4^, ^5^ However, the cloning efficiency is still very low (1%–5%), which restricts the technology's application.^2^, ^6^ Increasing numbers of studies have shown that many factors have impact on the efficiency of generating porcine and other mammalian SCNT‐derived embryos, which is measured with their developmental competences and quality. In this context, the crucial role is played by the epigenomic landscapes, as well as the genotypic and phenotypic traits associated with the tissue‐specific origin of nuclear donor cells and the degree of their differentiation or stemness.^7^, ^8^, ^9^, ^10^, ^11^, ^12^, ^13^ This, in turn, determines the ability of donor cell to reprogram their transcriptomic profiles within the cytoplasm of enucleated oocytes and within the blastomeres of resulting cloned embryos.^4^, ^12^, ^14^, ^15^, ^16^ Additionally, the quality of the nuclear recipient oocytes depends on factors such as synchronizing their meiotic, cytoplasmic, and epigenomic maturity states or artificially activating their embryo‐specific developmental programs.^17^, ^18^, ^19^ The capability of cloned embryos to progress beyond the developmental stages associated with the onset of embryonic genome transcriptional activity, known as maternal‐to‐embryonic transition (MET), is also significant.^17^, ^18^, ^19^, ^20^ Finally, the epigenetic programmability of donor cell genomes is influenced by inter‐transcriptomic and inter‐proteomic communication between the nuclear and mitochondrial compartments within SCNT‐derived embryo blastomeres.^21^ Taking those findings into consideration, it becomes evident that abnormal epigenetic modifications in donor cells are among the key factors hindering SCNT embryo development.^8^, ^14^, ^15^

N6‐methyladenosine (RNA m^6^A) is one of the most abundant RNA modifications in eukaryotic mRNAs.^22^ The m^6^A usually occurs in the context of RRACH (where R represents G or A, H represents A or C or U) sequences of RNAs and is mainly enriched in stop codons and 3′ untranslated region (UTR) mRNAs.^22^, ^23^ Similar to DNA methylation, RNA m^6^A levels are dynamic and reversible.^24^ The m^6^A modifications can be added by a series of methyltransferases (or ‘Writers’) or removed by demethylases (or ‘Erasers’).^24^ Furthermore, the m^6^A site is recognized by specific binding proteins (or ‘Readers’).^22^ RNA m^6^A methyltransferase complex (MTC) consists of METTL3, METTL14, WTAP, VIRMA, ZC3H13, CBLL1, and RBM15/15B.^25^ A large number of studies have demonstrated that RNA m^6^A modification exerts an impact on diverse processes such as embryo development, cellular reprogramming, gametogenesis, and disease formation by modulating RNA metabolism. This modulation encompasses mRNA stability, RNA export, degradation, translation, and alternative splicing.^24^, ^26^, ^27^, ^28^ In mouse embryonic fibroblasts, overexpression of ALKBH5 at the late stage promotes somatic cell reprogramming.^29^ Chen et al.^30^ found that gene‐ and cell‐type‐specific m^6^A mRNA modifications exist in different kinds of stem cells, and overexpressed METTL3 increases m^6^A abundance and promoted pluripotency factor expression, suggesting that m^6^A promotes somatic cell reprogramming. METTL3 knockdown decreases self‐renewal and triggers differentiation of porcine‐induced pluripotent stem cells (piPSCs) in RNA m^6^A dependent manner, and then blocks the transcription of KLF4 and SOX2.^31^ METTL14 has been found to affect early mouse embryogenesis by accelerating the change from naïve to primed state of the epiblast.^32^ Knock down of METTL5 decreased the development potentiality of SCNT embryos in mouse.^33^ All those studies suggest that RNA m^6^A modification may play important roles in cell‐fate transitions.^32^ However, the crucial role of RNA m^6^A and METTL14 during SCNT embryo development has not been clarified.

In this study, we compared RNA m^6^A level in two different types of donor cells and investigated the effects of the RNA m^6^A level of donor cells on the nuclear reprogramming of porcine SCNT embryos. Our results demonstrate that DNA methylation regulates RNA m^6^A modification by influencing the accessibility of transcription factor (TF) SP1 to the METTL14 promoter in donor cells. Furthermore, RNA m^6^A modification mediated by METTL14 regulates the stability of *TOP2B* mRNA and its protein translation via YTHDF1. Our study provides a new insight into the crosstalk between RNA m^6^A and DNA methylation, the most abundant modifications in porcine cells, and reveals the crucial role that RNA m^6^A modification plays in the developmental efficiency of SCNT embryos.

## 2 RESULTS

### 2.1 The characteristics of RNA m^6^A modification in two types of donor cells

**FIGURE 1:**
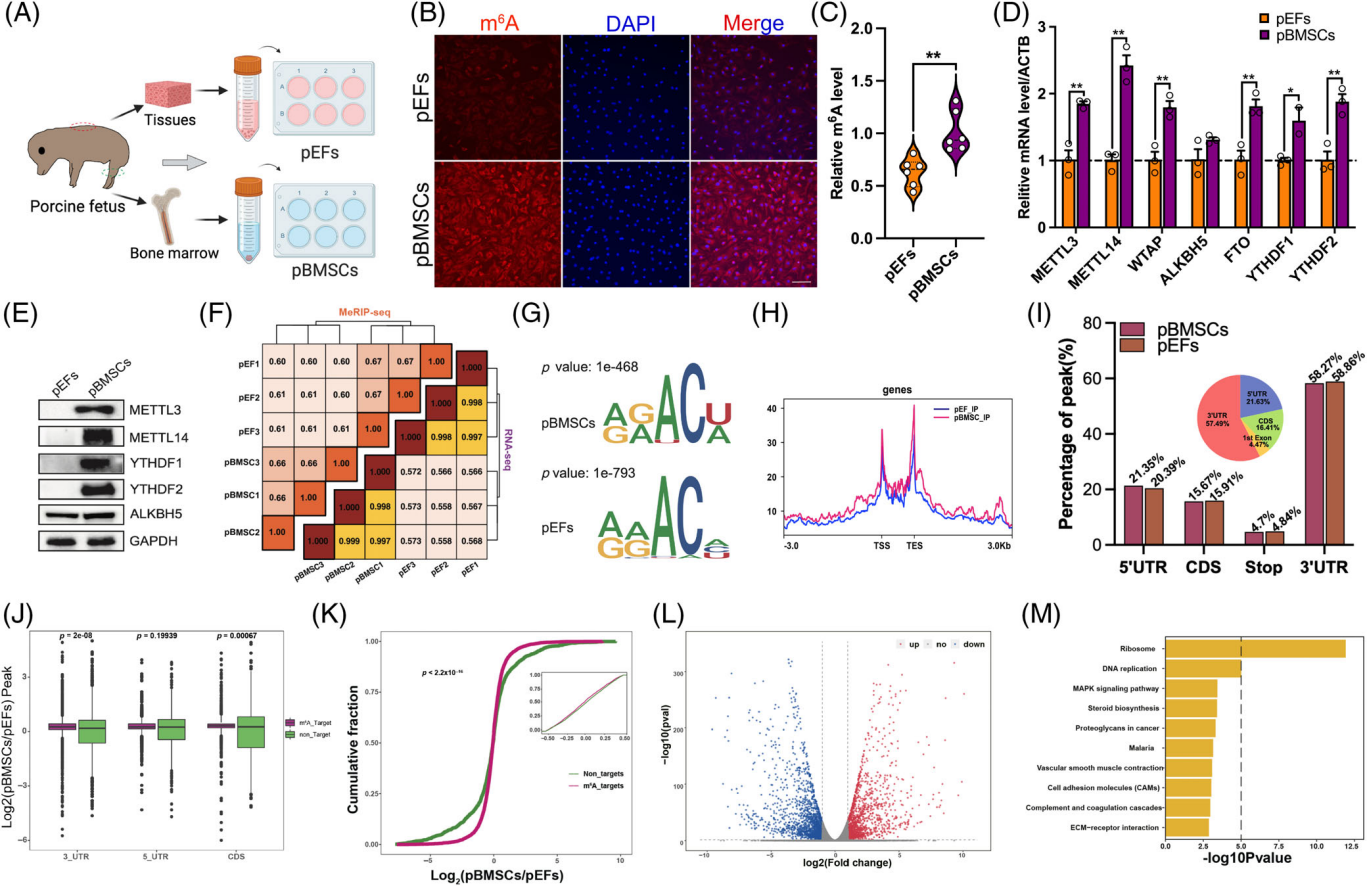
Global‐wide transcriptomic RNA N6‐methyladenosine (m^6^A) difference between different types of donor cells. (A) Primary porcine embryonic fibroblasts (pEFs) and porcine bone marrow mesenchymal stem cells (pBMSCs) were isolated from the skin tissues and bone marrow of porcine fetus. (B) Representative immunofluorescent (IF) staining images for RNA m^6^A (Red) and DAPI (Blue) from pEFs and pBMSCs. Scale, 300 *𝜇*m. (C) Total RNA m^6^A levels were quantified by colorimetric assay (*n* = 6). The (D) mRNA and (E) protein levels of RNA m^6^A‐related enzymes in different types of donor cells were analysed by qPCR. (F) The correlation analysis of methylated rna immunoprecipitation with next generation sequencing and RNA‐seq between different samples. (G) The consensus sequence signature of RNA m^6^A in two types of donor cells. (H) The distribution of m^6^A peaks in the whole genome regions. (I) The distribution of m^6^A peaks in different genome regions in two types of donor cells. The distribution of differentially methylated peaks between different types of donor cells was shown in pie chart. (J) The m^6^A fold changes of m^6^A_target genes and non_target genes in different genomic regions (5′ untranslated region [UTR], 3′ UTR and coding sequence). (K) Cumulative fraction of m^6^A_target and non_target genes in two types of donor cells. (L) Volcano plot of differentially expressed genes between the pBMSCs and pEFs. up, up‐regulated genes; down, down‐regulated genes; no, no difference between two groups. (M) Bar plot showing the top 10 enriched Kyoto encyclopedia of genes and genomes (KEGG) pathways. The presented data represent the mean of three independent experiments, with the error bars indicating the standard error of the mean (SEM). *p < 0.05, **p < 0.01.

Our previous observations revealed notable disparities in DNA methylation and histone modifications between two distinct types of porcine donor cells (bone marrow‐derived mesenchymal stem cells (BMSCs), porcine BMSCs (pBMSCs), and embryonic fibroblasts [pEFs]).^34^ These epigenetic modifications were found to influence the nuclear reprogramming of SCNT embryos.^34^ Based on these findings, we hypothesized that there might also be differences in RNA m^6^A modification between the two types of donor cells. To compare the RNA m^6^A modification levels between two types of donor cells, we isolated and cultured primary pBMSCs from porcine bone marrow and and pEFs from skin tissue (Figure 1A). Immunofluorescence (IF) staining results showed that the RNA m^6^A level in pBMSCs was higher than that in pEFs (Figure 1B). This finding was further confirmed using an enzyme‐linked immunosorbent assay (ELISA)‐based assay (*p* = 0.0025, Figure 1C). Quantitative polymerase chain reaction (qPC)R result suggested that the mRNA levels of several RNA m^6^A methylase‐related genes, including methyltransferase (METTL3, METTL14, and WTAP), demethylases (FTO), and RNA m^6^A Readers (YTHDF1 and YTHDF2) were significantly upregulated in pBMSCs than that in pEFs (*p* < 0.01, Figure 1D), the protein levels of these genes were confirmed by western blotting (Figure 1E). The above results demonstrated that pBMSCs has a higher RNA m^6^A level than pEFs.

To systematically investigate the global difference in RNA m^6^A modification between two types of donor cells, we conducted methylated RNA immunoprecipitation with next generation sequencing (MeRIP‐seq) in the present study. We found a strong correlation between the two datasets (MeRIP‐seq and RNA‐seq) within the same sample groups (Figure 1F). Additionally, a clear separation between pBMSCs and pEFs datasets was observed, as depicted in the correlation heatmap (Figure 1F). The consensus sequence of m^6^A modification was GGACU in the pBMSCs group, and AAACA in the pEFs group (Figure 1G). These findings are consistent with the classical RRACH RNA m^6^A motif in pigs.^22^ The RNA m^6^A modification was predominantly observed around the transcription start site (TSS) and transcription end site. We observed a higher global transcriptomic RNA m^6^A level in pBMSCs compared with pEFs, which was consistent with the RNA m^6^A levels detected by IF and ELISA assays (Figure 1H). Moreover, we conducted an analysis of the distribution of RNA m^6^A sites across the entire genome. The results showed that the majority of RNA m^6^A sites (~58%) were located in the 3′ UTRs, followed by the 5′ UTR region (~20%; Figure 1I). For each individual gene, it was observed that ~90% of genes possessed no more than five RNA m6A sites. Furthermore, the different m^6^A sites were predominantly located in the 3′ UTR, 5′ UTR, and coding sequence (CDS) regions (Figure 1I). Furthermore, mRNAs (m^6^A_Target) containing different RNA m^6^A peaks in their CDS (*p* = 0.00067) and 3′ UTR (*p* = 2 × 10^−8^) regions exhibited higher expression levels compared with mRNAs (non_Target) without such distinct RNA m^6^A peaks (*p* < 0.05, Figure 1J). At the global transcription level, genes with RNA m^6^A modification exhibited lower mRNA levels in pBMSCs compared with pEFs (*p* < 2 × 10^−16^, Figure 1K). There were 1657 downregulated genes and 1477 upregulated genes in pBMSCs compared with pEFs (Figure 1L). The KEGG pathway analysis revealed that the differentially methylated genes (DMGs) were primarily enriched in pathways such as Ribosome, Proteoglycans in cancer, MAPK signalling pathway, and ovarian steroidogenesis pathway (Figure 1M). These results suggest that there are substantial differences in RNA m^6^A modification between pBMSCs and pEFs, which may affect the development efficiency of SCNT embryos.

### 2.2 DNA methylation affected TF SP1 binding to METTL14 promoter

**FIGURE 2:**
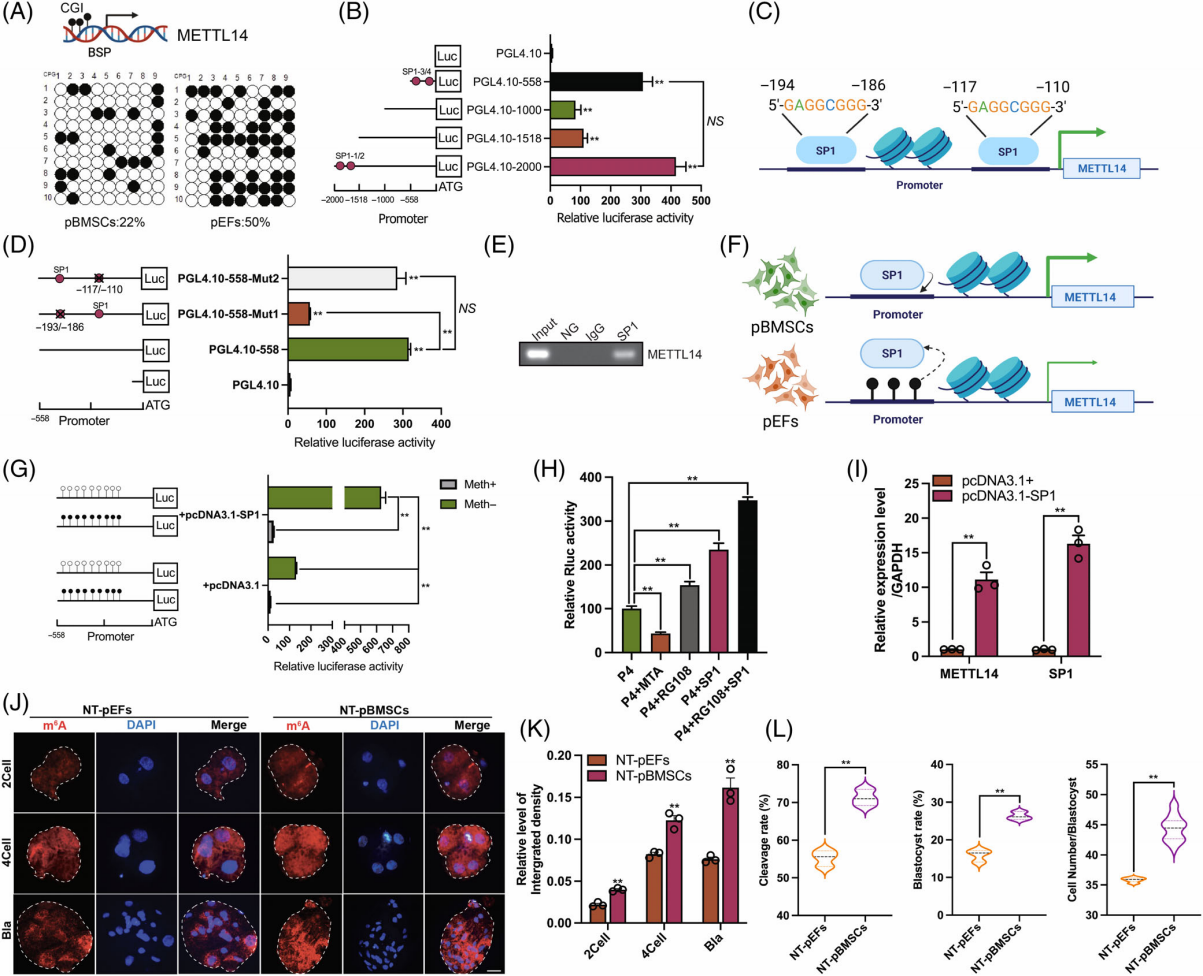
Promoter hypo‐methylation promotes transcription factor SP1 binding with METTL14. (A) Bisulphite sequencing PCR assay was performed to compare the DNA methylation level in METTL14 promoter between porcine bone marrow mesenchymal stem cells (pBMSCs) and porcine embryonic fibroblasts (pEFs). (B) The luciferase activity of different promoter regions of METTL14. (C) Two potential SP1 binding sites in porcine METTL14 promoter. (D) The luciferase activity of the core promoter region of METTL14 with or without SP1 binding sites mutation. (E) Chromatin immunoprecipitation (ChIP) assay was performed to identify the binding of SP1 and the core promoter region of METTL14. Input, before immunoprecipitation sample; No antibody control group (NG), sample without antibody; immunoglobulin G (IgG), sample was incubated with IgG antibody; SP1, sample was incubated with SP1 antibody. (F) Schematic diagram shown that DNA methylation affects the binding of SP1 with METTL14 promoter in two types of donor cells. (G) The luciferase activity of the METTL14 promoter with or without DNA methylation, SP1 overexpression. (H) The luciferase activity of the METTL14 promoter treated with MTA, RG108, or SP1 overexpression. (I) The relative mRNA level of METTL14 and SP1. (J) Representative IF staining images for RNA N6‐methyladenosine (m^6^A) (Red) and DAPI (Blue) in somatic cell nuclear transfer (SCNT) embryos derived from different donor cells. (K) The relative integrated density of RNA m^6^A in different development stages. (L) The cleavage and blastocyst rate between SCNT embryos derived from different donor cells. The data represent the mean of three independent experiments ±SEM. *NS*, *p* > 0.05, *p < 0.05, **p < 0.01.

DNA methylation has recently been discovered to interact with RNA m^6^A in various physiological and pathological processes.^35^, ^36^ To investigate whether DNA methylation mediates RNA m^6^A deposition during cell reprogramming, we initially analysed the CpG island in several RNA m^6^A‐related enzymes. Interestingly, we found a CpG island in the promoter region of METTL14 (Figure S1), an RNA m^6^A ‘Reader’ in RNA MTC, which was previously found to be differentially expressed between porcine SCNT and in vivo developed (IVV) embryos.^37^ Bisulphite sequencing PCR (BSP) results indicated that the DNA methylation level of the METTL14 promoter was lower in pBMSCs compared with pEFs (22% vs. 50%; Figure 2A). We then conducted a luciferase activity assay to validate the core promoter region of METTL14 (Figure 2B). The results showed that the region spanning −558 to +1 bp (PGL4.10‐558) served as the core promoter region of METTL14 (Figure 2B). Previous studies have indicated that DNA methylation at specific TF‐binding sites within gene promoter regions physically obstructs the binding of TF complexes to DNA, thereby regulating gene expression.^38^ We discovered the presence of four TF SP1 binding sites, with two located within the −558/+1 bp region and the other two within the −2000/−558 bp region (Figure 2C). Moreover, our findings revealed that these two closely situated SP1 binding sites (−194/−186 bp, −118/−110 bp) were located within the core promoter region of METTL14. Introducing mutations in the first SP1 binding site (−117/−110 bp, PGL4.10‐558‐Mut1; *p* = 0.0043), but not in the second SP1 binding site (−193/−186 bp, PGL4.10‐558‐Mut2; *p* = 0.066), led to a significant reduction in luciferase activity compared with the wild‐type (WT) core promoter (*p* < 0.05, Figure 2D). Remarkably, we observed that the SP1 binding site at −194/−186 bp exhibited high conservation across pigs, mice, and humans (Figure S2). Furthermore, the species‐conserved binding of SP1 to the METTL14 promoter was also identified through published chromatin immunoprecipitation sequencing (ChIP‐seq) data.^27^, ^28^ The binding of SP1 to the porcine METTL14 promoter was further confirmed through ChIP assay in pEFs (Figure 2E). Based on our findings, we proposed that the hypermethylated status could hinder the binding of SP1 to the METTL14 promoter, resulting in lower transcriptional activity in pEFs compared with pBMSCs (Figure 2F). The METTL14 promoter activity was assessed in donor cells. Our result indicated that DNA methylation significantly reduced the METTL14 promoter activity in vitro (*p* = 0.0024, Figure 2G). Compared with the control group, the overexpressed of SP1 resulted in an increased METTL14 promoter activity (*p* = 0.0001, Figure 2G). However, whether SP1 was overexpressed or not, DNA methylation hindered the promoter activity of METTL14 (Figure 2G). Additionally, the promoter activity of METTL14 was significantly decreased when donor cells were treated with SP1 inhibitor MTA (*p* = 0.0073, Figure 2H). Conversely, METTL14 promoter activity was upregulated in donor cells treated with the DNA methylation inhibitor RG108 (*p* < 0.016, Figure 2H). Furthermore, we observed that both SP1 overexpression alone and in combination with RG108 significantly enhanced the promoter activity of METTL14 (Figure 2H). This finding was consistent with the qPCR results, which showed a significant increase in *METTL14* expression upon SP1 overexpression (*p* < 0.001, Figure 2I). Taken together, our findings verify that DNA methylation influences the binding of the TF SP1 to the METTL14 promoter in donor cells.

To compare the developmental efficiency of SCNT embryos derived from two types of donor cells (pEFs and pBMSCs), we proceeded to construct SCNT embryos (Figure 2J). As expected, RNA m^6^A level was significantly higher in the NT‐pBMSCs group than in the NT‐pEFs group during the early stages of development, (including the two‐cell, four‐cell), and blastocyst stages (*p* < 0.001, Figure 2K). In comparison to the NT‐pEFs group, the NT‐pBMSCs group exhibited higher rates of cleavage (71% ± 1.25% vs. 55.27% ± 1.22%), blastocyst formation (26.33 ± 0.62 vs. 15.93 ± 0.94), and cell number (44.44 ± 0.47 vs. 35.85 ± 0.12) in the SCNT embryos (*p* < 0.001, Figure 2L).

### 2.3 DNA methylation regulates RNA m^6^A modification through METTL14 in donor cells

**FIGURE 3:**
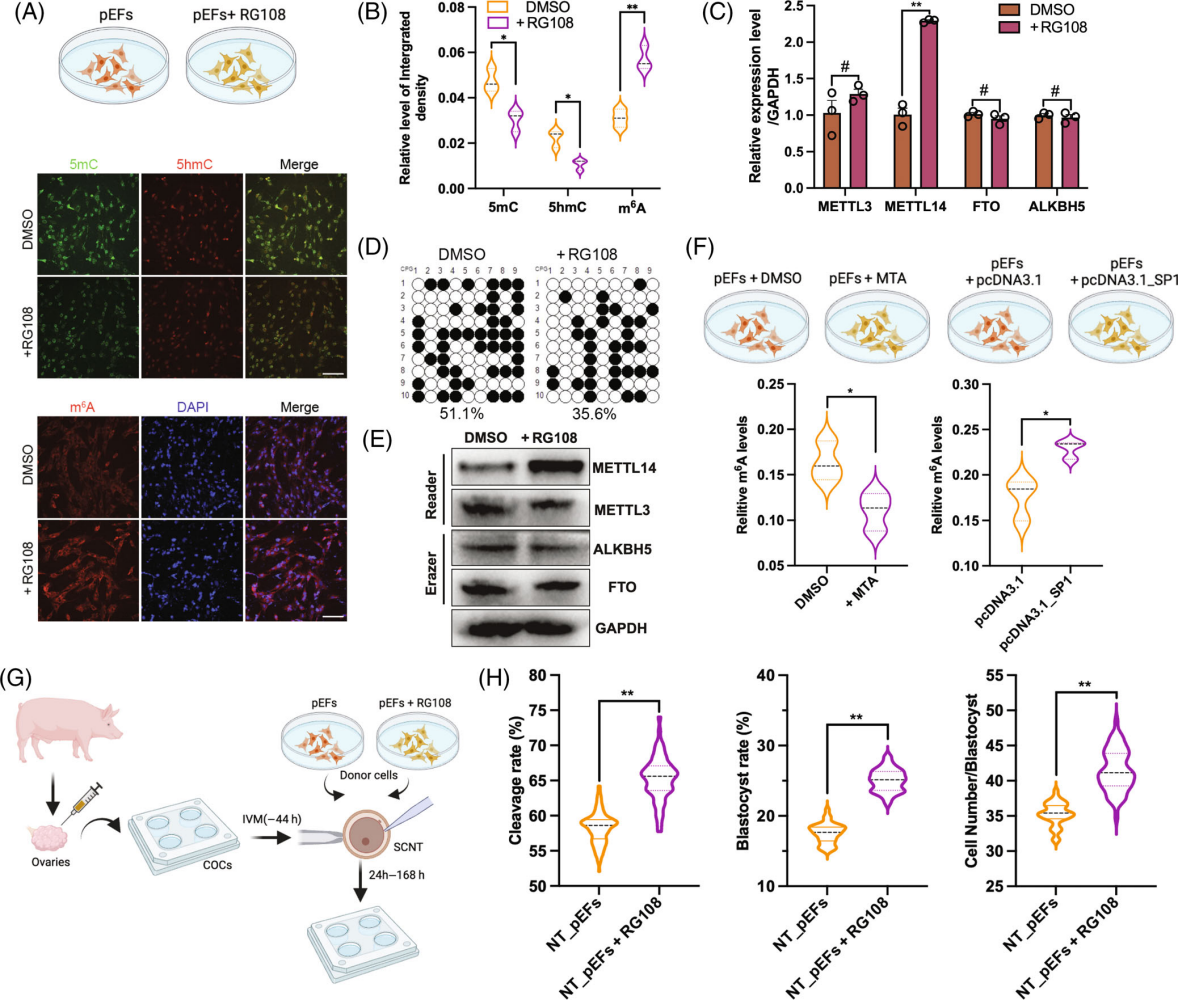
Inhibit DNA methylation promotes RNA N6‐methyladenosine (m^6^A) through METTL14. (A) Representative IF staining images for 5mC (green), 5hmC (red), RNA m^6^A (red), and DAPI (blue) in porcine embryonic fibroblasts (pEFs) treated with or without RG108. (B) The relative integrated density of 5mC, 5hmC, RNA m^6^A in two groups. (C) qPCR of METTL3, METTL14, FTO, and ALKBH5. (D) DNA methylation level of METTL14 promoter region was analysis by bisulphite sequencing PCR (BSP) assay in pEFs treated with DMSO or RG108. (E) Western blot of METTL3, METTL14, FTO and ALKBH5 in pEFs treated with DMSO or RG108. (F) Relative m^6^A level of pEFs treated with MTA or overexpressed SP1. (G) Schematic diagram of the somatic cell nuclear transfer (SCNT) derived from pEFs treated with RG108 or not (DMSO). (H) Statistical analysis of cleavage, blastocyst rate and cell number per blastocyst. The data represent the mean of three independent experiments ±SEM. *NS*, *p* > 0.05, *p < 0.05, **p < 0.01. IVM, in vitro maturation.

To investigate the regulation of DNA methylation on RNA m^6^A, we isolated the total RNA and protein from pEFs treated with 20 μM RG108 for 48 h (Figure 3A). IF staining results revealed that RG108 treatment significantly reduced the level of 5‐methylcytosine (5mC, *p* = 0.024) and 5‐hydroxymethylcytosine (5hmC, *p* = 0.022), while increasing the level of RNA m^6^A (Figure 3B, *p* = 0.004). Furthermore, qPCR results indicated that RG108 treatment resulted in an upregulation of *METTL14* mRNA levels (Figure 3C, *p* = 0.002). However, no significant changes were seen in the expression levels of other RNA m^6^A‐related enzymes (Figure 3C, *p* > 0.05). Subsequently, we compared the DNA methylation levels of the METTL14 promoter. As expected, the DNA methylation level exhibited a significant decrease in the RG108‐treated group compared with the dimethyl sulfoxide (DMSO)‐treated group (Figure 3D, 51.1% vs. 35.6%). Western blotting analysis revealed an increase in the METTL14 protein levels upon RG108 treatment (Figure 3E). Furthermore, the total RNA m^6^A level was found to be diminished in donor cells treated with SP1 inhibitor MTA (Figure 3F, *p* < 0.036), while it was increased in donor cells overexpressing SP1 (Figure 3F, *p* = 0.02). Subsequently, we evaluated the developmental efficiency using SCNT technology (Figure 3G). Compared with the control group, SCNT embryos derived from donor cells treated with RG108 exhibited a significant increase in the cleavage rate (65.2% ± 3.2% vs. 58.3% ± 2.2%, *p* = 0.0011), blastocyst rate (25.12% ± 2.1% vs. 17.78% ± 1.3%, *p* < 0.001), and cell number of blastocyst (42.2% ± 2.7% vs. 35.3% ± 1.4%, *p* < 0.001; Figure 3H). These findings are consistent with our previous study.^39^ Together, these results suggest that SP1 directly binds to METTL14 promoter, and the METTL14 expression is regulated by DNA methylation, thereby affecting the RNA m^6^A level in donor cells.

### 2.4 The global transcriptomic effect of METTL14 overexpression in donor cells

**FIGURE 4:**
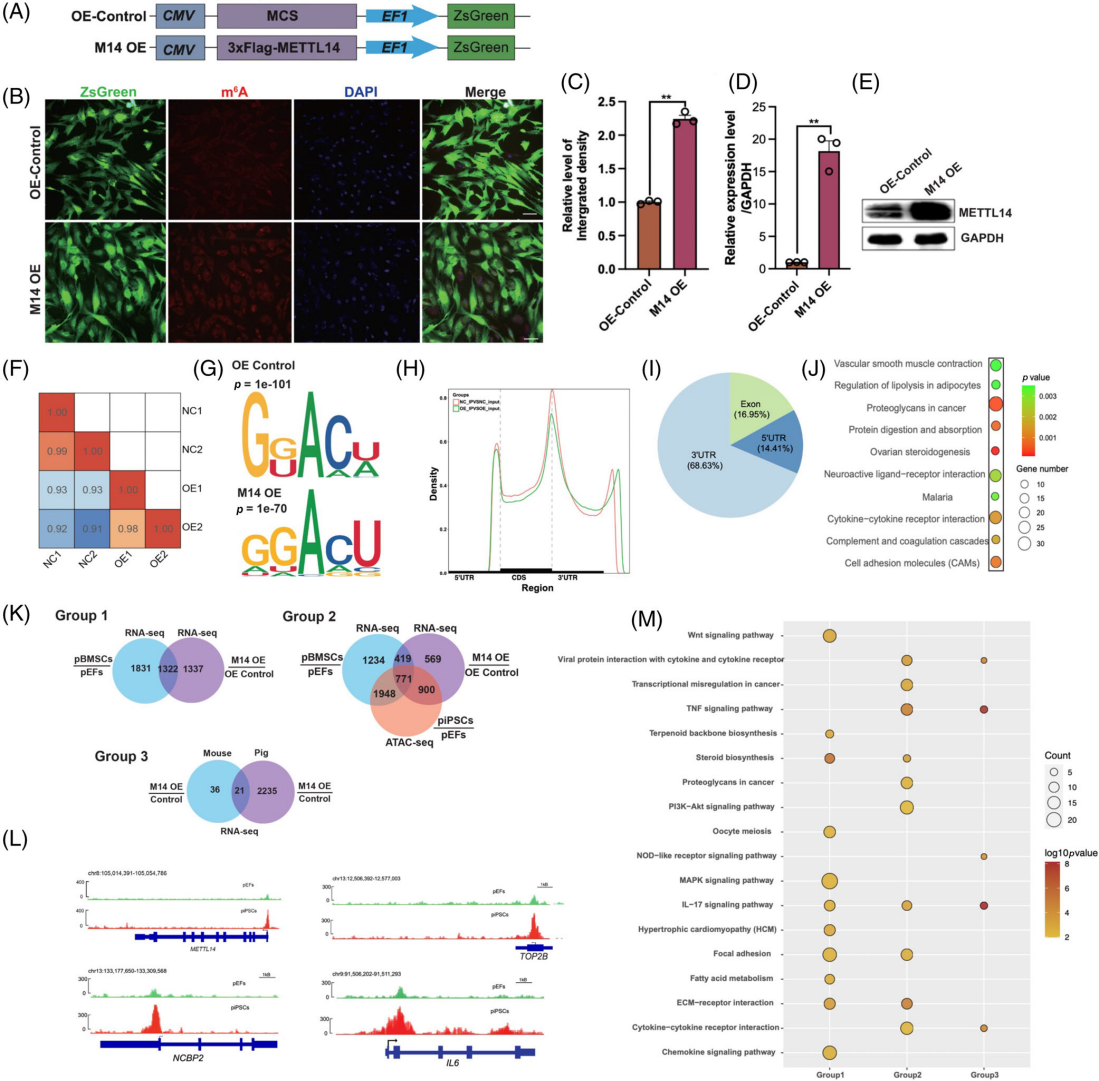
Transcriptomic wide effects of METTL14 overexpression in porcine embryonic fibroblasts (pEFs). (A) Structure of the Control (OE‐Control) and METTL14 overexpression (M14 OE) lentiviral vector. (B) Immunofluorescence (IF) staining for RNA N6‐methyladenosine (m^6^A; red) and nuclei (DAPI), ZsGreen (green). (C) Relative integrated density of RNA m^6^A in pEFs with or without METTL14 overexpression. (D) qPCR of METTL14 in control and METTL14 overexpression groups. (E) Western blot of METTL14 in control and METTL14 overexpression groups. (F) Correlation analysis of RNA‐seq. (G) Consensus RNA m^6^A motif in two groups. (H) RNA m^6^A peak density distribution in genome regions (5′ untranslated region [UTR], coding sequence [CDS], 3′ UTR). (I) Genome region analysis of differentially RNA m^6^A peak. (J) Pathway enrichment analysis of differentially expressed genes. (K) Venn plots for different comparisons. (L) integrative genome viewer browser visualization of the ATAC‐seq peak within several genes. (M) Pathway enrichment analysis between three comparisons groups.

To better understand, the crucial role of METTL14 during SCNT embryo development, we performed lentiviral infection to overexpress porcine METTL14 in pEFs (Figure 4A). IF straining results showed that METTL14 overexpression significantly upregulated RNA m^6^A level (Figure 4B,C, *p* = 0.01). The overexpression effect of METTL14 in donor cells was confirmed by both qPCR and Western blotting analysis (*p* = 0.003, Figure 4D,E). Furthermore, we employed MeRIP‐seq and RNA‐seq technologies to investigate the comprehensive transcriptomic effects of METTL14 overexpression. As shown in Figure 4F, the correlation coefficient between samples within the same group was higher (*r* > 0.98) compared with samples from different groups (*r* < 0.93). The consensus sequence of RNA m^6^A modification sites in control group was found to be GG(or U) ACU(or A) in, while in M14 OE‐group it was GGACU in M14 OE‐group (Figure 4G). These findings are consistent with previous studies in pigs.^40^ The distribution of RNA m^6^A peak was primarily observed in mRNA transcripts, spanning from TSS to the 3′ UTR region (Figure 4H). The differential m^6^A modification peaks were primarily located in the 3′ UTR (68.63%), followed by Exon (16.95%) and 5′ UTR (14.41%; Figure 4I). Functional enrichment analysis was performed on the DMGs, revealing several significantly enriched pathways such as Proteoglycans in cancer, ovarian steroidogenesis, Cytokine–cytokine receptor interaction, and others (*p* < 0.001, Figure 4J).

Previous study has found that chromatin accessibility regions played important role in nuclear reprogramming.^29^ To gain further insights into the transcriptomic changes during nuclear reprogramming, we conducted an integrated analysis of RNA‐seq and published ATAC‐seq data in our study. We identified a total of 1322 differentially expressed genes (DEGs) that were shared between different types of donor cells and METTL14 overexpression or non‐overexpression in donor cells (Group 1). Among them, a subset of 771 genes showed distinct changes in chromatin accessibility during the process of nuclear reprogramming (Group 2). Upon METTL14 overexpression in donor cells, we found identified 21 DEGs that were shared between mice and pigs (Group 3; Figure 4K,I). Interestingly, the ATAC‐seq data revealed a significant increase in chromatin accessible at the METTL14 locus in piPSC compared with pEFs (Figure 4I), suggesting that METTL14 may play a crucial role during the process of nuclear reprogramming.^41^ Furthermore, we observed that genes enriched in senescence‐associated secretory phenotype (SASP) pathways in both Groups 2 and 3, including cytokine and cytokine receptor interaction, and IL‐17 signalling pathway were found (Figure 4M). This suggests that METTL14 overexpression transiently enhanced the expression of SASP genes, which have been implicated as key factors in regulating reprogramming efficiency.^42^, ^43^

### 2.5 Donor cells RNA m^6^A level affected the development ability of porcine SCNT embryos

**FIGURE 5:**
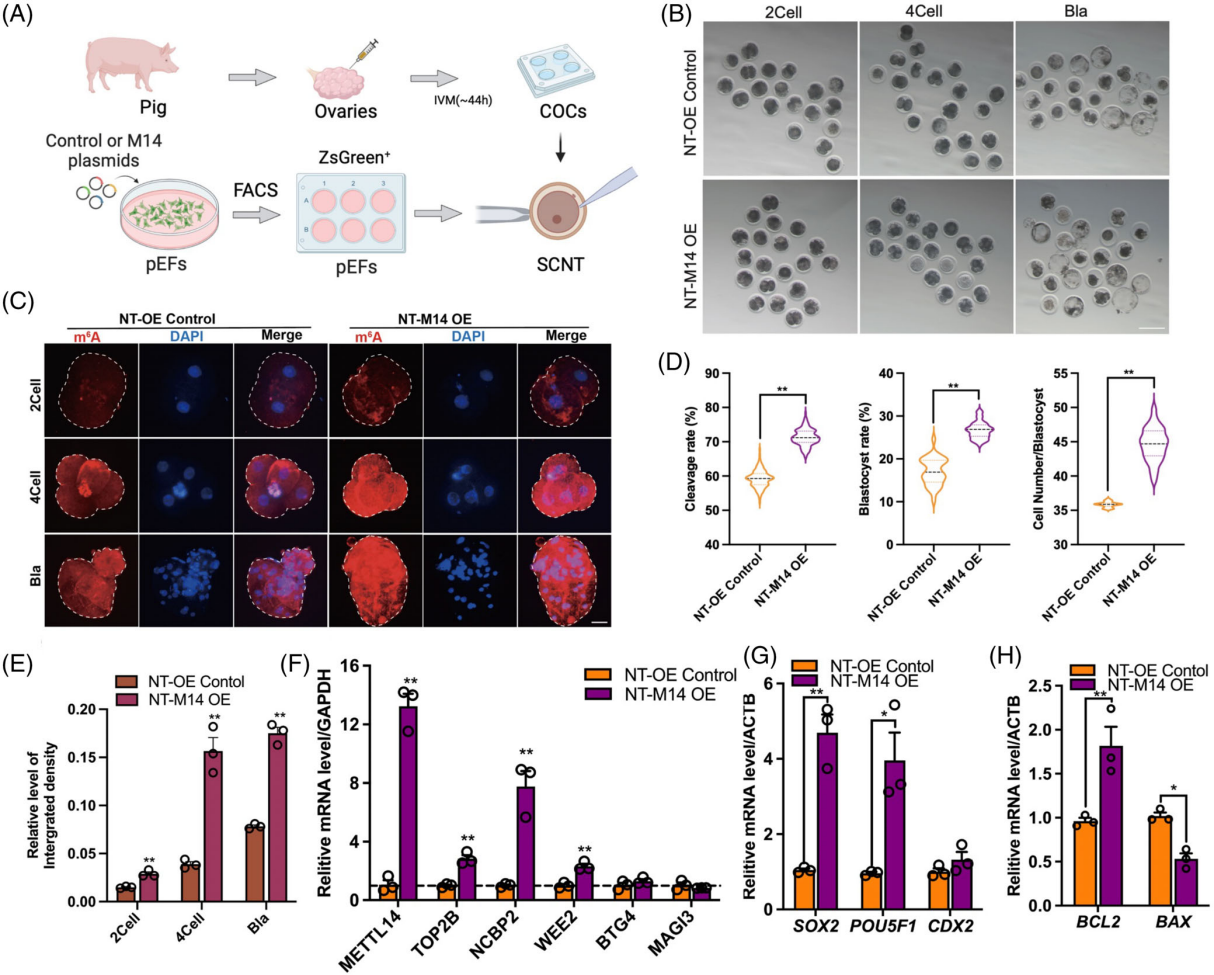
The effect of METTL14 overexpression in donor cells on the somatic cell nuclear transfer (SCNT) embryos development. (A) Schematic diagram of SCNT derived from donor cells (porcine embryonic fibroblasts [pEFs]) with or without METTL14 overexpression. (B) Development status of SCNT embryos at different time points. (C) Representative IF staining images for RNA N6‐methyladenosine (m^6^A, red) and DAPI (blue) of SCNT embryos derived from METTL14‐overexpression‐donor cells or control cells during early embryos development. (D) Statistical analysis of cleavage, blastocyst rate and cell number per blastocyst. (E) The relative integrated density of RNA m^6^A in different development stages among different groups. (F) The relative mRNA levels of several genes in SCNTs embryos at the stage of four‐cell. (G) The relative mRNA levels of pluripotency genes and (H) apoptosis‐related genes. The presented data represent the mean of three independent experiments, with the error bars indicating the standard error of the mean (SEM). *p < 0.05, **p < 0.01. COCs, cumulus–oocyte complexes.

We generated SCNT embryos using donor cells (pEFs) with (NT‐M14 OE) or without METTL14 overexpression (NT‐OE‐Control; Figure 5A). Compared with the control group, the METTL14 overexpression group showed a significant increase in the cleavage rate (71.3% ± 2.4% vs. 59.4% ± 2.7%, *p* = 0014), blastocyst rate (27.0% ± 1.9% vs. 16.8% ± 2.5%, *p* = 0021), and cell number in blastocyst (45.25% ± 2.15% vs. 35.86% ± 0.41%, *p* < 0001) in porcine SCNT embryos (Figure 5B,D). We performed an IF staining assay to detect the RNA m^6^A levels during SCNT embryo development. The results suggested that METTL14 overexpression (NT‐M14 OE) significantly increased the RNA m^6^A levels at the two‐cell, four‐cell, and blastocyst stages of SCNT embryo development (Figure 5C,E, *p* < 0.001). qPCR result indicated that METTL14 overexpression (NT‐M14 OE) led to a significant increase in the mRNA abundance of several candidate genes (Figure 5F, *p* < 0.05), and the pluripotent genes such as *SOX2* and *POU5F1*, as well as anti‐apoptosis‐related genes *BCL2* (Figure 5G,H, *p* < 0.001).

**FIGURE 6:**
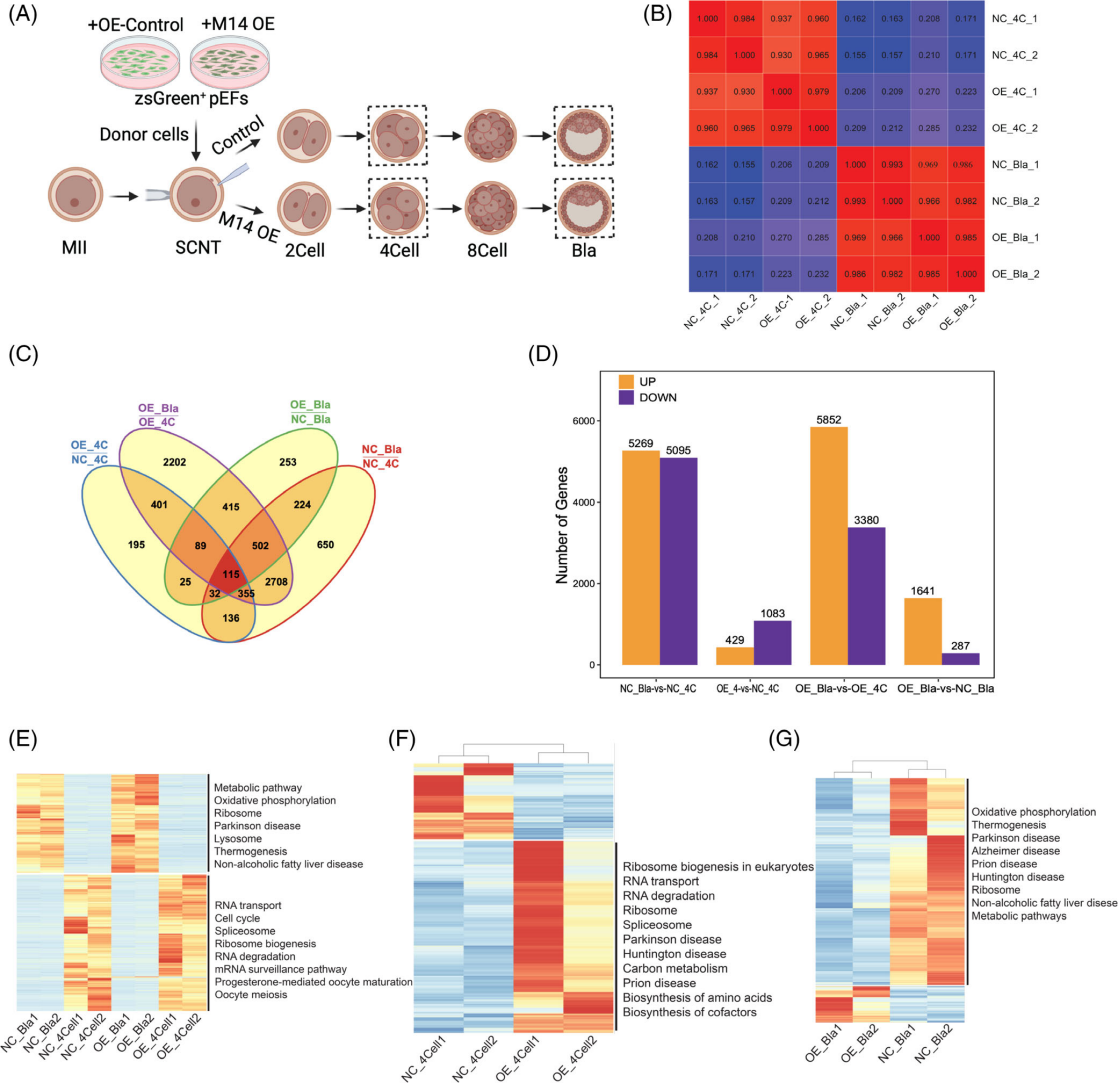
Transcriptome‐wide effect of METTL14 overexpression in donor cells on somatic cell nuclear transfer (SCNT) embryos. (A) Schematic diagram shown the RNA‐seq. Dashed boxes indicate samples used for RNA‐seq. (B) The correlation analysis of RNA‐seq between different samples. (C) Veen plot of DEGs between different groups. (D) Bar plot of up‐regulated and down‐regulated genes between different groups. (E) Heatmap of differentially expressed genes (DEGs) between four groups. DEGs between SCNT embryos derived from METTL14‐over‐expression‐donor cells or control cells at (F) four‐cell and (G) blastocyst stages. KEGG pathways were shown in the right panel of heatmaps.

We then performed the RNA‐seq for the SCNT embryos with (OE) or without (NC) METTL14 overexpression in donor cells (Figure 6A). As expected, we observed a higher correlation coefficient between samples derived from the same group compared with samples from different groups in the RNA‐seq analysis (Figure 6B). The analysis revealed that there were 429 upregulated genes and 1083 downregulated genes at the four‐cell stage, while there were 1641 up‐regulated genes and 287 down‐regulated genes at the blastocyst stage (NT_Bla; Figure 6C,D). Those DEGs at the four‐cell stage were primarily enriched in metabolic pathways, ribosome biogenesis in eukaryotes, and RNA transport pathways (Figure 6E,F). At the blastocyst stage, DEGs were primarily enriched in metabolic pathways, lysosome, and protein processing in the endoplasmic reticulum (Figure 6G). We integrated our previous transcriptome data from SCNT and IVV embryos with the present data and identified several intersection DEGs, including NCBP2, TOP2B, WEE2, and BTG4, which are known to be involved in RNA metabolism. This suggests that these genes can be regulated by RNA m^6^A modification and may play important roles in early embryo development and the development efficiency of SCNT embryos.

**FIGURE 7:**
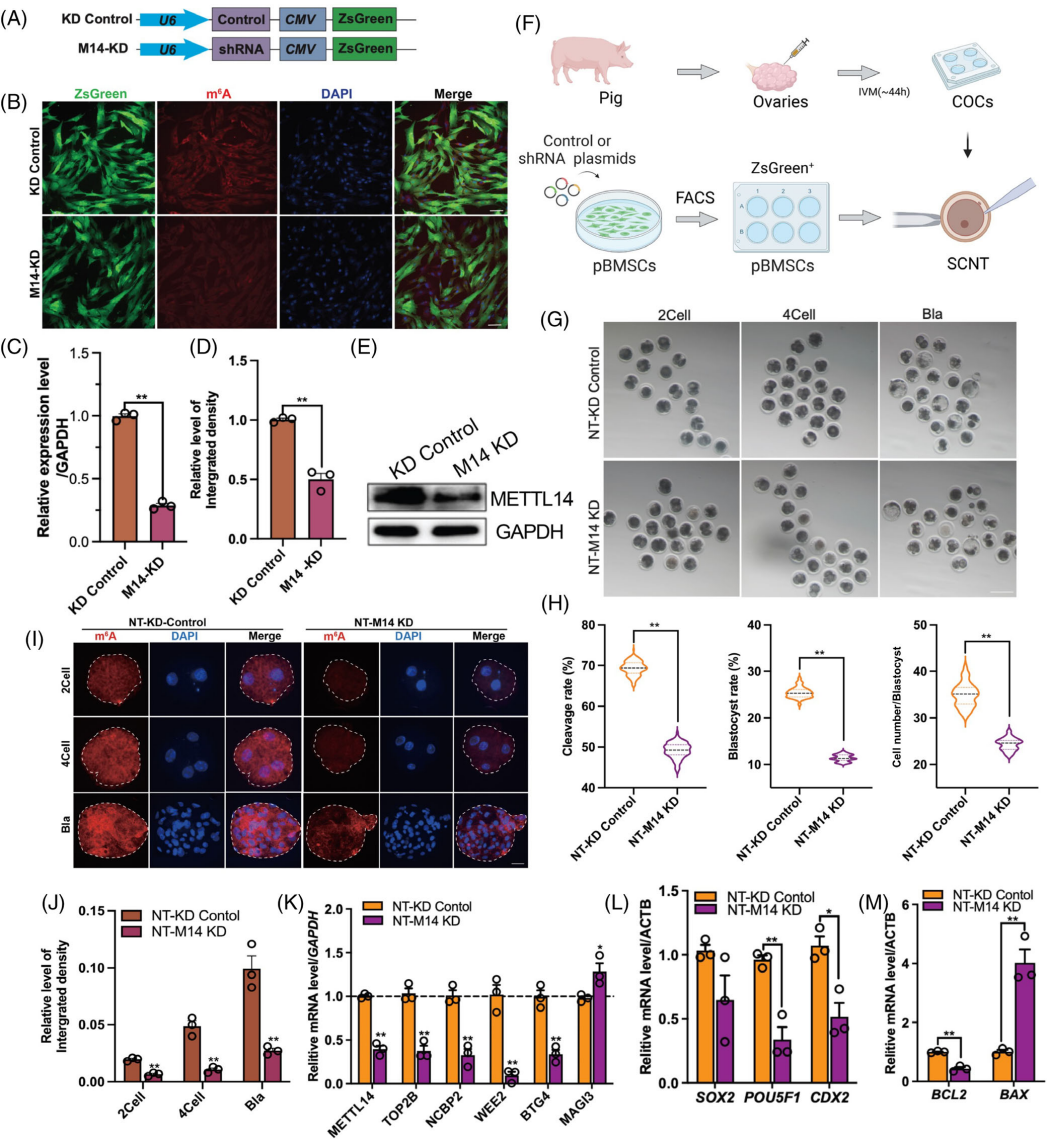
The effect of METTL14 knockdown on RNA N6‐methyladenosine (m^6^A) and development efficiency in somatic cell nuclear transfer (SCNT) embryos. (A) Schematic diagram of lentivirus plasmid structure. (B) Representative IF staining results of RNA m^6^A (red) and nuclei (DAPI); ZsGreen (green). (C) The relative integrated density of RNA m^6^A, (D) mRNA levels and (E) protein level between two groups. (F) Schematic diagram of the production of SCNTs embryos with METTL14 knockdown in donor cells. (G) Development status of SCNT embryos at different time points. (H) Statistical analysis of cleavage, blastocyst rate and cell number per blastocyst. (I) Representative immunofluorescence (IF) staining images for RNA m^6^A (red) and DAPI (blue) of SCNT embryos derived from METTL14‐overexpression‐donor cells or control cells during early embryos development. (J) The relative integrated density of RNA m^6^A in between two groups. (K) The relative mRNA levels of several genes in SCNTs embryos at the stage of four‐cell. (L) The relative mRNA levels of pluripotency‐ genes and (M) apoptosis‐related genes. The presented data represent the mean of three independent experiments, with the error bars indicating the standard error of the mean (SEM). *p < 0.05, **p < 0.01. COCs, cumulus–oocyte complexes.

We then constructed lentivirus for METTL14 knockdown (Figure 7A). IF staining showed that METTL14 knockdown (M14 KD) significantly reduced the RNA m^6^A levels in pBMSCs (Figure 7B,C). The knockdown efficiency was further confirmed by qPCR and Western blotting analysis (Figure 7D,E). Furthermore, knockdown of the METTL14 in donor cells resulted in a significant decrease in the developmental efficiency of SCNT embryos, including a lower cleavage rate (69.4% ± 1.7% vs. 49.5% ± 2.1%, *p* < 0.001), blastocyst rate (25.3% ± 1.1% vs. 11.0% ± 0.6%, *p* = 0.003), and a reduced cell number in blastocysts (34.52% ± 2.21% vs. 24.24% ± 1.14%; Figure 7F–H, *p* < 0.001). Similar to the observation in donor cells, knockdown of METTL14 led to a significant decrease in RNA m^6^A levels at early development (two‐cell, four‐cell, and blastocyst stages) stages of SCNT embryos (Figure 5I,J, *p* < 0.001). Moreover, compared with the control group (NT‐KD‐Control), METTL14 knockdown (NT‐M14 KD) resulted in a lower abundance of several candidate genes, pluripotent gene *POU5F1* (*p* = 0.011), and lineage differentiation‐related factor *CDX2* (*p* = 0.024), as well as anti‐apoptosis‐related genes *BCL2* (*p* = 0.004), while there was a higher abundance of apoptosis‐related *BAX* (Figure 7K–M, *p =* 0.005). These findings highlight the critical role of METTL14 in regulating gene expression during the early development of SCNT embryos.

### 2.6 RNA m^6^A modification regulated mRNA stability and translation of TOP2B via YTHDF1

**FIGURE 8:**
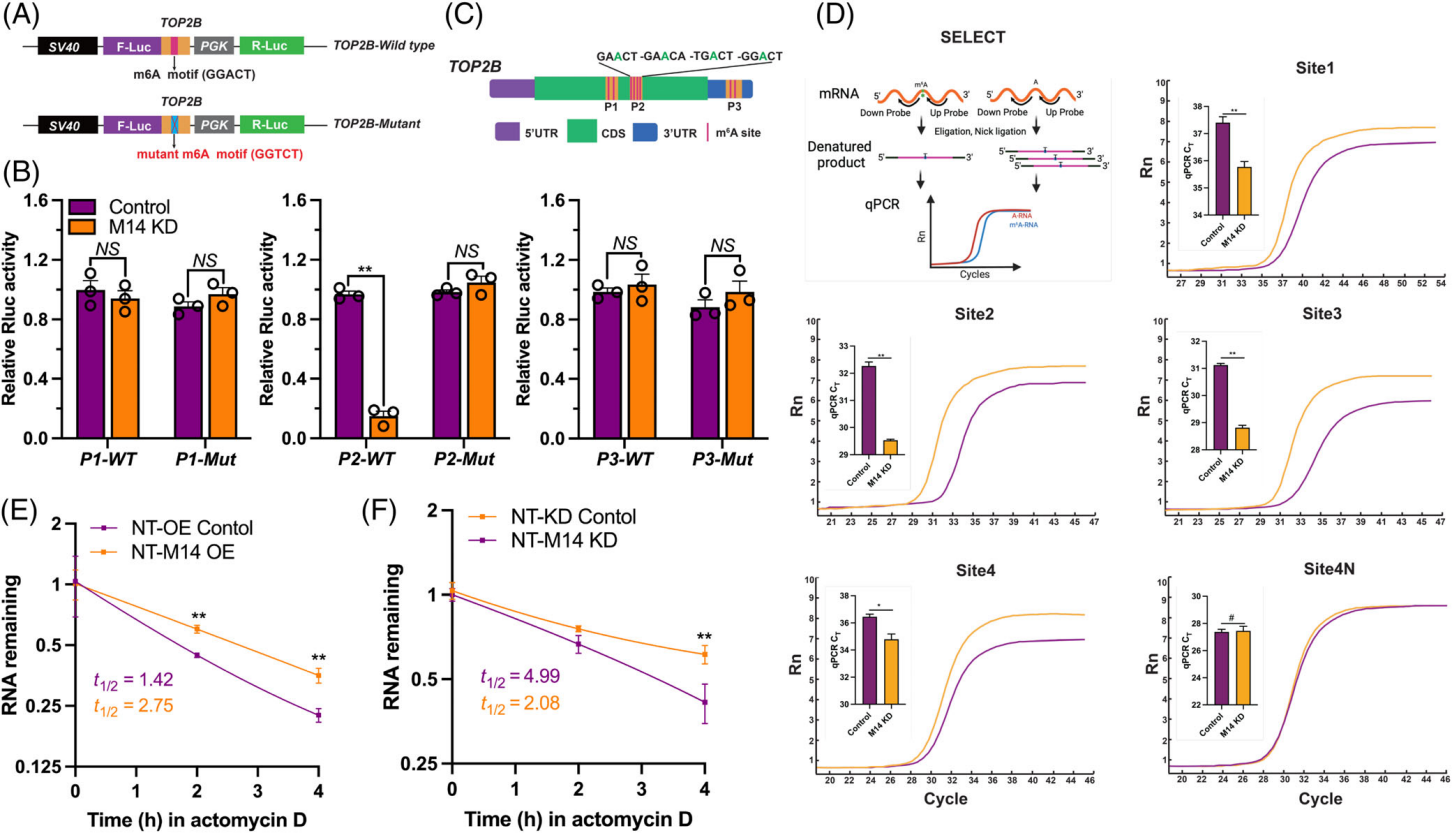
The regulates of METTL14 on the specific RNA N6‐methyladenosine (m^6^A) sites of TOP2B mRNA. (A) Schematic diagram of the location of RNA m^6^A sites in TOP2B mRNA. (B) Construction of the plasmid for RNA m^6^A detection. (C) Relative Rluc activity of wild‐type and mutant groups. (D) SELECT assay for specific m^6^A sites in TOP2B mRNA. (E) The effect of METTL14 overexpression or (F) knockdown on TOP2B mRNA decayed rate. The presented data represent the mean of three independent experiments, with the error bars indicating the standard error of the mean (SEM). *p < 0.05, **p < 0.01.

DNA topoisomerase II beta (TOP2B) is a type of DNA topoisomerase that plays crucial roles in various biological processes, including relieving the supercoiling of DNA, maintaining chromosome structure, regulating DNA topology, and participating in DNA replication and repair.^44^, ^45^ Since TOP2B exhibited differential expression between porcine SCNT embryos derived from different donor cells, we subsequently investigated the regulatory mechanism involving RNA m^6^A in TOP2B expression. Utilizing the SRAMP online software, we identified several potential RNA m^6^A sites within the *TOP2B* mRNA sequence. A WT vector and a mutant vector, in which the m^6^A site was replaced with a T nucleotide, were constructed for *TOP2B* mRNA (Figure 8A). We generated three pairs of reporters (P1, P2, and P3) with WT and mutant (Mut) sequence to investigate the potential RNA m^6^A site (Figure 8B). Our results showed that knockdown of METTL14 (M14 KD) significantly suppressed the luciferase activity of the P2–WT reporter, indicating that METTL14 was involved in the m^6^A methylation of *TOP2B* mRNA. In contrast, the luciferase activity of the P2–Mut reporter, which lacked the m^6^A site, was not affected by METTL14 knockdown (Figure 8C). These findings support the role of METTL14 in mediating the m^6^A methylation of *TOP2B* mRNA. Given the presence of four potential RNA m^6^A sites in the P2 reporter, we performed a SELECT assay to identify the specific RNA m^6^A sites in TOP2B mRNA. The results of the SELECT assay showed that the knockdown of METTL14 (M14 KD) inhibited the ligation of probes across the four different RNA m^6^A sites in *TOP2B* mRNA. This suggests that METTL14 is involved in the methylation of these specific m^6^A sites in *TOP2B* mRNA, and its knockdown leads to a reduction in m^6^A modification at these sites (Figure 8D).

To further investigate the regulation mechanism of RNA m^6^A and TOP2B, we examined the mRNA stability of *TOP2B* in SCNT embryos derived from Control and M14 KD‐donor cells. Our results indicated that the mRNA level of *TOP2B* was significantly increased in the NT‐M14 OE group compared with the control group after treatment with Actinomycin D (Figure 8E, *p* < 0.001). Conversely, the stability of *TOP2B* mRNA in SCNT embryos derived from M14 KD‐donor cells (NT‐M14 KD) was significantly lower compared with the NT‐KD Control group (Figure 8F, *p* = 0.009). These findings suggest that RNA m^6^A plays a regulatory role in the stability of *TOP2B* mRNA during porcine SCNT embryo development.

### 2.7 Knockdown of TOP2B decreased the development ability of porcine SCNT embryos

**FIGURE 9:**
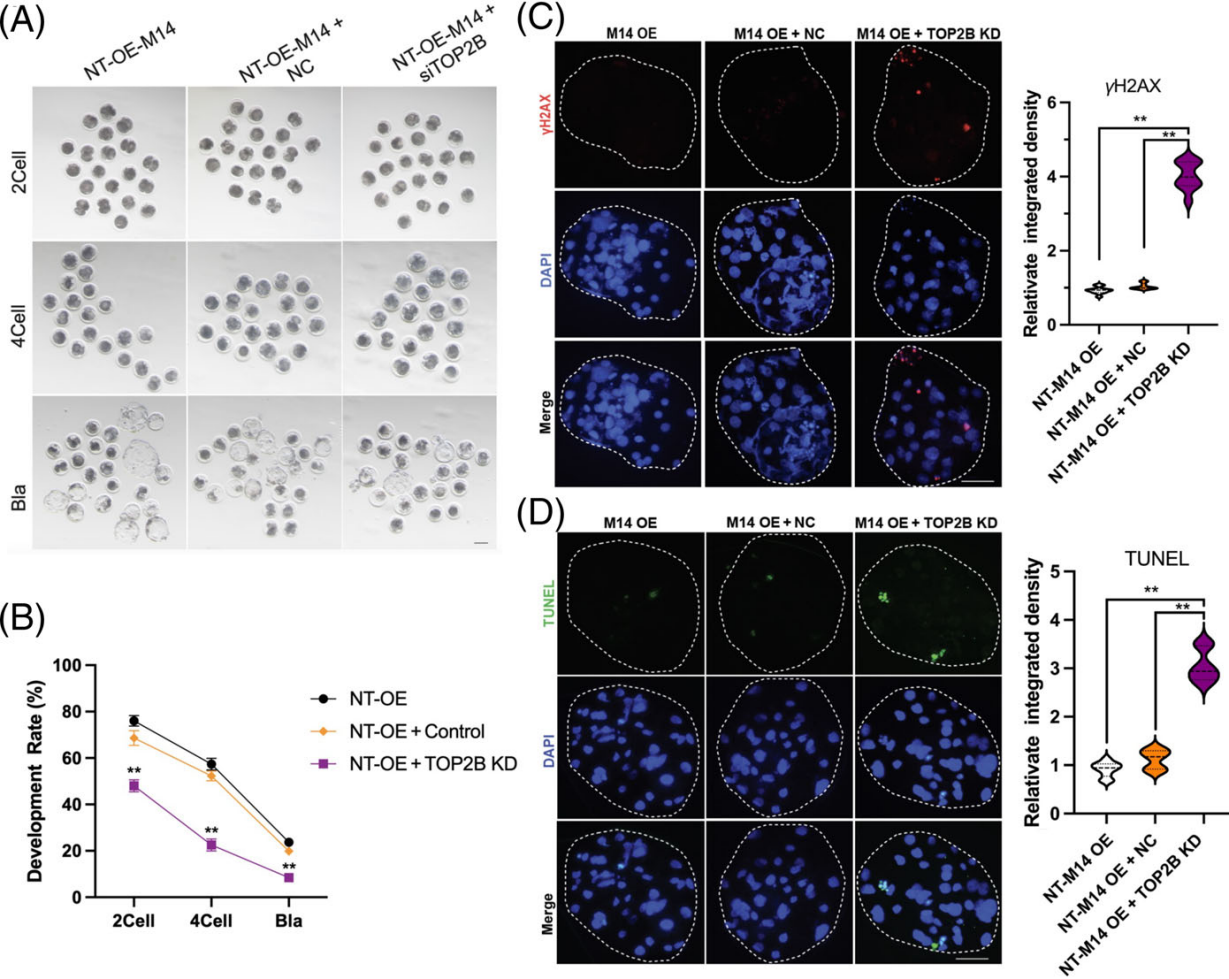
The effect of TOP2B knockdown on the quality of somatic cell nuclear transfer (SCNT) embryos. (A) Development status and rate of SCNT embryos after TOP2B KD. (C) Representative immunofluorescence (IF) staining and relative integrated density analysis for γH2AX (red) and DAPI (blue). (D) Representative IF staining and relative integrated density analysis for TUNEL (green) and DAPI (blue). The presented data represent the mean of three independent experiments, with the error bars indicating the standard error of the mean (SEM). *p < 0.05, **p < 0.01. UTR, untranslated region.

To further investigate the function of TOP2B in SCNT embryos, we performed TOP2B knockdown by microinjecting *TOP2B* siRNA into SCNT embryos derived from donor cells with or without METTL14 overexpression (Figure 9A). Our results showed that *TOP2B* knockdown significantly impaired the cleavage (*p* < 0.001) and blastocyst rates (*p* = 0.003) of SCNT embryos compared with SCNT embryos derived from donor cells with METTL14 overexpression (Figure 9B). All those results suggested that knockdown of TOP2B hindered the promotional effect of METTL14 overexpression in donor cells during the development of SCNT embryos. Furthermore, IF staining revealed that *TOP2B* knockdown significantly increased the DNA damage signal phospho‐histone H2A.X (γH2AX) at the blastocyst stage of SCNT embryos compared with the negative control groups (Figure 9C, *p* < 0.001). Additionally, *TOP2B* knockdown led to an increased rate of cell apoptosis at the blastocyst stage of SCNT embryos (Figure 9D, *p* < 0.01). These findings suggest that the expression level of TOP2B has a significant impact on the development efficiency of SCNT embryos.

### 2.8 YTHDF1 knockdown decreased the development ability of porcine SCNT embryos

**FIGURE 10:**
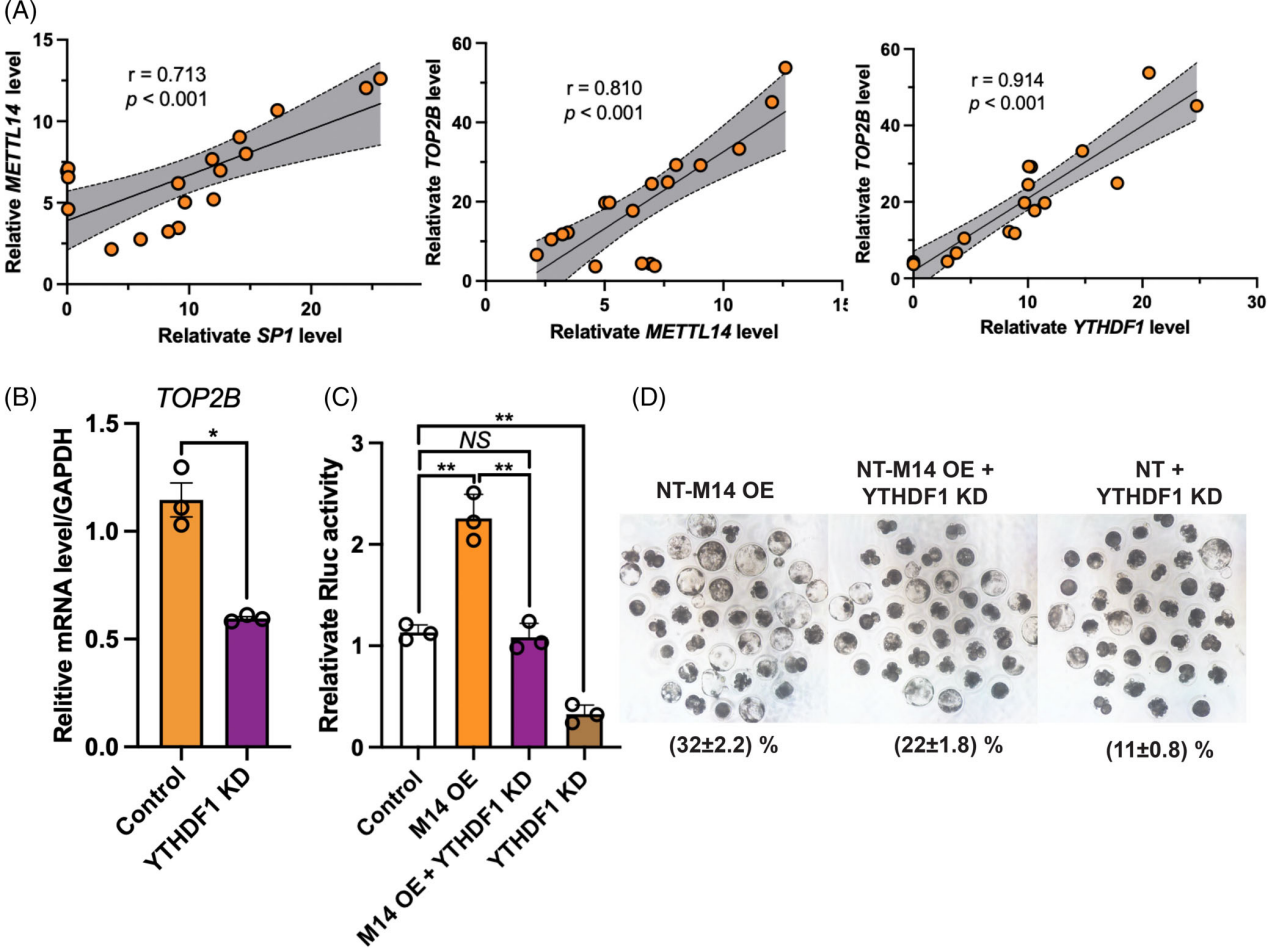
The SP1‐METTL14‐YTHDF1‐TOP2B pathway during somatic cell nuclear transfer (SCNT) embryos development. (A) The correlation analysis between SP1 and METTL14, METTL14 and TOP2B, and YTHDF1 and TOP2B. (B) The relative mRNA level of TOP2B after YTHDF1 knockdown. (C) The relative Rluc activity between different groups. (D) The effect of YTHDF1 KD on SCNT embryos development.

YTHDF proteins have been reported to play crucial roles in somatic cell reprogramming.^46^ We then turned our attention to the RNA m^6^A reader YTHDF1, which had been identified as a key player in embryo development. To gain a better understanding of the molecular regulatory mechanism involving SP1, YTHDF1, METTL14, and TOP2B during SCNT embryo development, we examined their interrelationships. Our result showed a significant positive correlation between the mRNA levels of SP1 and METTL14 (*r* = 0.713, *p* < 0.001), as well as between TOP2B (*r* = 0.810, *p* < 0.001), and YTHDF1 (*r* = 0.914, *p* < 0.001) in porcine embryos (Figure 10A). qPCR analysis revealed that knockdown of YTHDF1 resulted in a downregulation of *TOP2B* mRNA levels during the development of SCNT embryos (*p* < 0.025, Figure 10B). Compared with the control group, overexpression of METTL14 significantly increased the luciferase activity of the TOP2B‐WT reporter, indicating its positive regulation on TOP2B expression (Figure 10C), On the other hand, knockdown of YTHDF1 significantly inhibited the luciferase activity of the TOP2B‐WT reporter (*p* = 0.004, Figure 10C), suggesting its role in promoting TOP2B expression. In addition, we observed that overexpression of METTL14 partially increased the luciferase activity of the TOP2B‐WT reporter (*p* = 0.01, Figure 10C). Finally, we utilized the M14‐OE pBMSC as donor cells for SCNT embryo generation. Consistent with the luciferase activity results, we observed a significant increase in the blastocyst rate of SCNT embryos derived from M14‐OE donor cells compared with the control group. On the contrary, knockdown of YTHDF1 significantly reduced the blastocyst rate of SCNT embryos, while the blastocyst rate could be partially rescued by overexpressing METTL14 in donor cells (Figure 10D).

## 3 DISCUSSION

SCNT technology, which has been developed and applied in various species for many years, plays important roles in animal breeding and the construction of disease models.^47^, ^48^, ^49^ However, the development efficiency of porcine SCNT embryo is extremely low compared with other species.^2^ During the development of SCNT embryos, epigenetic modification undergoes extensive rearrangement to promote zygotic genome activation (ZGA).^50^ Previous study has shown that RNA m^6^A modification may involve in the aberrant reprogramming in porcine SCNT embryos.^51^ In this study, we found that there was a substantial difference in RNA m^6^A level between different types of donor cells. An increase in m^6^A abundance promotes reprogramming of mouse embryo fibroblasts to pluripotent stem cells, while a decrease in m^6^A levels hinders reprogramming.^30^ This suggests that RNA m^6^A modification differs between different types of cells and that altering m^6^A modification can affect cell reprogramming. Since BMSCs belong to pluripotent stem cells with certain differentiation potential,^52^ while fibroblast cells belong to terminally differentiated somatic cells,^53^ the difference of RNA m^6^A modification levels between the two types of cells can also affect reprogramming efficiency. Compared with pEFs, SCNT embryos derived from pBMSCs showed a significantly improved development efficiency, suggesting RNA m^6^A level of the donor cells may play an important role in somatic cell reprogramming. During the development of SCNT embryos, it is well studied that RNA m^6^A modification is involved in the degradation of maternal mRNA or activation of the zygotic genome.^54^, ^55^ It is necessary for certain levels of RNA m^6^A modification to be maintained in donor cells to promote the degradation of maternal genes or activation of the zygotic genome.^56^, ^57^, ^58^

Increasing evidence has shown that there exists crosstalk between RNA m^6^A and other epigenetic modifications (DNA methylation, H3K36me3, H3K27me3, H3K27ac, H3K4me3, H3K9me2/3) during various physiological and pathological process.^25^, ^36^, ^59^, ^60^, ^61^, ^62^ As a most extensively modifications, DNA methylation plays a role in controlling gene expression, maintaining chromatin condition, and ensuring genome stability^35^. A recent study has shown that METTL3‐mediated RNA m^6^A modification could regulate DNA methylation and chromatin accessibility in human.^35^ Yang et al.^38^ found that DNA methylation affects the expression of the IGF2BP3 by affecting the accessibility of SP1 then regulates muscle development. Here, we reported DNA methylation impeded TF SP1 binding to the METTL14 promoter region, thereby regulated RNA m^6^A modification in donor cells, subsequently affected development of embryo derived from SCNT embryos. We speculated that lower RNA m^6^A level may be due to high DNA methylation level inhibiting the transcription of the METTL14 in pEFs. Then, we confirmed that DNA methylation affected the binding of SP1 in the promoter region and thereby regulated METTL14 expression. Our result showed that donor cells treated with RG108, a DNMTs inhibitor,^63^ simultaneously increased the RNA m^6^A modification levels as well as the development efficiency of SCNT embryos, which is consistent with our previous report^34^. Therefore, based on the results of this study, we believe that RG108, a well‐known DNA demethylating agent, can not only facilitate nuclear reprogramming but also establish a crosstalk between RNA m^6^A and DNA methylation during SCNT embryo development. However, further research is needed to fully understand the effect of DNA methylation on global RNA m^6^A modification.

Given the observed difference in the development efficiency of SCNT embryos derived from these two types of donor cells,^55^ we hypothesize that the RNA m^6^A levels in donor cells may influence the nuclear reprogramming process of SCNT embryos. In this study, overexpressed METTL14 increased RNA m^6^A level in donor cells and to some extent improve the efficiency of embryo development derived from SCNT. Conversely, decreasing the level of m^6^A modification in donor cells can inhibit the SCNT embryo development, indicating that abnormal RNA m^6^A modification in SCNT embryos may affect reprogramming and embryonic development.^64^ This is consistent with recent studies in somatic cell reprogramming in iPSCs and embryonic stem cells.^42^, ^65^ Despite some similarities and differences in the reprogramming mechanisms of SCNT and iPSCs,^29^, ^46^ METTL14 may play a broad and conserved role in overcoming epigenetic barriers based on our present study. In addition, Li et al. report that RNA m^6^A modification may reduce H3K9me2 modification, another recently study shows that METTL14 knockout increases H3K27me3 level, thus decreasing global gene expression.^65^ The improvement of SCNT embryonic development caused by the increase of METTL14 may also be related to regulation function in an m^6^A‐independent manner,^42^, ^66^, ^67^ such as the decrease of H3K9me2 and H3K27me3 levels but need to be further verified.^35^ Additionally, whether m^6^A‐mediated non‐coding RNAs play an important role in SCNT embryos still needs to be explored.

Abnormal maternal gene degradation can cause embryonic development stopping at the zygotic stage, while abnormal ZGA is the main reason that affects the development of SCNT embryos in mammals.^37^, ^50^ In our previous study, we identify that a large number of ribosome‐ and variable cleavage‐related genes are significantly up‐regulated at the four‐cell stage by single‐cell RNA‐seq (scRNA‐seq) technology compared with two‐cell stage, indicating that transcription regulation plays a huge role in this stage of the embryos.^37^ Overexpression of METTL14 in donor cells affected the gene expression related to metabolic pathways, ribosome biosynthesis, cleavage bodies, RNA degradation signalling pathways in SCNT embryos. Interestingly, Long et al.^51^ report that several genes, including *METTL14*, *NCBP2*, and *KDM4B* were significantly reduced in blocked SCNT embryos in mice, indicating that abnormal epigenetic modifications and incomplete activation of transcription pathways are potential molecular barriers in SCNT embryos. In our study, we noticed that many genes related to metabolism were differentially expressed at the four‐cell and blastocyst stages, and these DEGs at the blastocyst stage were found to be mainly enrich in metabolic pathways and signalling pathways, such as oxidation and phosphorylation. This suggests that changes in RNA m^6^A levels may be involved in the metabolic processes underlying SCNT embryo development.

TOP2B, as a DNA topoisomerase, is mainly involved in chromosomal condensation, DNA transcription, and replication, and plays an important role in the separation of sister chromatids during mitosis.^44^, ^45^ It can regulate and change the topological state of DNA and promote double‐strand break repair.^45^ On the other hand, TOP2B interacts with cohesions and CTCF at the topological domain boundary, thereby promoting rapid gene expression during early embryonic development.^68^ Our result showed that an increase in m^6^A levels led to an enhancement in the stability of *TOP2B* mRNA, while knocking down RNA m^6^A reduced TOP2B protein level, indicating that RNA m^6^A was involved in the regulation of TOP2B expression. Therefore, we speculated that RNA m^6^A modification mediated TOP2B regulation may play a key role in gene expression and chromosomal topological structure regulation during early embryonic development.

DNA damage has been found during oocyte aging and early embryo development.^69^, ^70^ As a DNA topoisomerase, TOP2B is significantly decreased in mature oocytes of elderly individuals, knockdown of TOP2B causes embryos to be blocked at the two‐cell stage.^70^ These studies indicate that a disruption in the expression ratio of TOP2B is detrimental to early embryo development. Additionally, DNA damage is also an important marker of blastocyst quality, and it has been shown that RNA m^6^A plays a critical role in the ultraviolet‐induced DNA damage repair process.^71^ In porcine parthenogenetic embryo, knocking down WTAP decreases the overall m^6^A modification level and pluripotency gene expression in blastocysts, while also reduces blastocyst rates and increases the number of apoptotic cells, indicating a relationship between RNA m^6^A modification and DNA damage in embryos.^72^ In this study, we found that knocking down *TOP2B* increased DNA damage signals, suggesting that TOP2B may affect SCNT embryonic development by influencing DNA damage and genomic stability.

**FIGURE 11:**
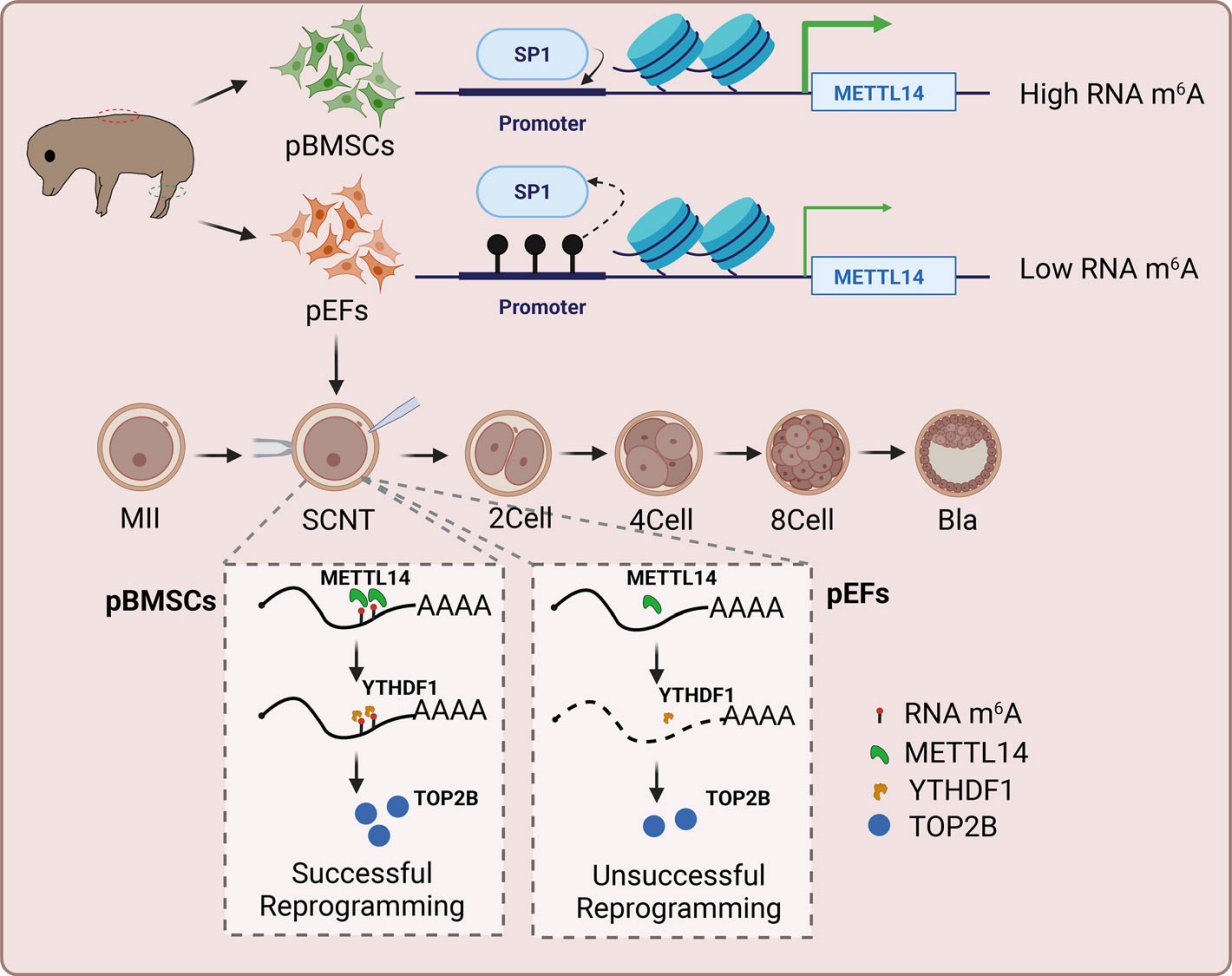
Schema of the crosstalk between DNA methylation‐RNA N6‐methyladenosine (m^6^A) in the regulation of porcine somatic cell nuclear transfer (SCNT) embryo development. pBMSCs, porcine bone marrow mesenchymal stem cells; pEFs, porcine embryonic fibroblasts; SCNT, somatic cell nuclear transfer.

In conclusion, this study demonstrates that different type of donor cells has different levels of RNA m^6^A modification and therefore has different effects on nuclear reprogramming and developmental ability of SCNT embryos. The DNA methylation/SP1/RNA m^6^A/TOP2B axis highlights an important role of crosstalk between RNA m^6^A modification and DNA methylation during SCNT embryo development. A higher level of RNA m^6^A modification mediated by METTL14 improves development efficiency of SCNT embryo by increasing the DNA damage and genomic stability (Figure 11). These data highlight the crucial role of SP1‐METTL14‐mediated RNA m^6^A modification regulates the mRNA stability of TOP2B via YTHDF1 during nuclear reprogramming of SCNT embryos. Our study gives a better understanding of crosstalk between epigenetic modifications and SCNT embryo development.

## 4 MATERIALS AND METHODS

### 4.1 In vitro maturation of porcine oocytes

The porcine ovaries were collected at a local abattoir. Ovaries were immediately transported to the laboratory within 2–4 h at 37°C in 0.9% NaCl buffer supplemented with 5% penicillin–streptomycin. Cumulus–oocyte complexes (COCs) were collected using a 10 mL syringe, and 200 COCs with a minimum of three layers were chosen for further experimentation. COCs were cultured in in vitro maturation (IVM) medium for 22–44 h at 38.5°C, 5% CO_2_, and 95% air. Subsequently, IVM medium was changed to another hormone‐free maturation medium (with the same composition as the earlier IVM medium, excluding epidermal growth factor, luteinizing hormone, and foollicle‐stimulating hormone). They were maintained in this medium for a duration of 20 h at 38.5°C, under conditions of 5% CO_2_ and 95% air. The separation of cumulus cells from COCs was facilitated using hyaluronidase at a concentration of 0.2%. The oocytes displaying first polar body extrusion were considered mature and were chosen for subsequent experimental procedures.

### 4.2 Preparation and treatment of donor cells

The pBMSCs were isolated and cultured followed by our previous studies.^34^, ^73^ Briefly, the foetus (E30–E35) was collected and placed in 75% ethanol for 5 min and then washed three times with phosphate‐buffered saline (PBS). The bone marrow suspension was flushed by Dulbecco's modified eagle medium (DMEM)/F12 (Gibco, Waltham, MA) using a 10 mL syringe. The suspension was washed with prewarmed DMEM/F12 medium, centrifuged at 1000 rpm for 5 min, and discarded the supernatant. The cell pellet was re‐suspended in DMEM/F12 medium supplemented with 10% foetal bovine serum (Gibco) and 0.1% penicillin/streptomycin (v/v). Subsequently, the suspension was incubated at 37°C under a 5% CO_2_ environment. After an initial culture duration of 8 h, the existing medium was substituted with fresh medium. When pBMSCs reached up to 90% confluent, The cells (~1 × 10^6^) were subjected to passaging for subsequent experiments. The identification and multi‐lineage differentiation of pBMSCs was performed followed by our previous study.^73^

The pEFs were isolated according to our previous description.^34^ Briefly, the tissues were cut into 1 mm^3^‐fragments. The sample was exposed to a solution containing 0.25% trypsin and incubated at 37°C while being gently shaken for a duration of 20 min. Then DMEM was added to quench the digestion, and cells were centrifugated at 1000 rcf for 5 min. The cell pellet was re‐suspended in a complete DMEM medium and then seeded onto a cell culture dish at 37°C, 5% CO_2_. Cells (~1 × 10^6^) were passaged or frozen when reached up to 90% confluent.

To conduct plasmid transfection, cells at ~80% confluence were transfected with the desired plasmid using the FuGENE® HD transfection reagent (Promega, Madison, USA). For the drug treatment, 200 nM MTA (Selleck Chemicals, TX, USA) and 20 μM RG108 (Sigma, MO, USA) or DMSO were added to medium for 48 h for the following experiment.

### 4.3 Somatic cell nuclear transfer

SCNT was executed in accordance with our previously protocol.^34^ Briefly, the blind‐suction method was used to separate the nucleus from oocytes under inverted microscope (Nikon, Japan). The well‐prepared donor cells were directly injected into the enucleated MII oocytes. Then the 200 reconstructed embryos were incubated in porcine zygote medium 3 (PZM3) for 1 h before following treatment. Subsequently, the reconstructed embryos (15 at a time) were washed twice with fusion medium and then activated using two direct‐current pulses at 1.2 kV/cm for 30 μs using an ECM2001 electro‐fusion instrument (BTX, Holliston, MA). Check and select successfully reconstructed embryos after a 30‐min incubation in CO_2_ incubator following fusion and discard the non‐fused embryos. Fifty reconstructed embryos were cultured in 50‐μL PZM3 medium at 38.5°C for 7 days.

### 4.4 MeRIP‐seq and bioinformatics analysis

Total RNA was extracted from 5 × 10^8^ donor cells by using Trizol reagent (Invitrogen, CA, USA). The concentration and purity of the extracted RNA were determined using a NanoDrop ND‐1000 instrument (NanoDrop, DE, USA). The integrity of the RNA samples was assessed using a Bioanalyzer 2100 (Agilent, CA, USA). A sample of total RNA with a concentration >50 ng/μL, RIN >8.0, OD260/280 >1.8, and an amount >50 μg was considered for the following experiment. Oligo(dT)‐beads (Ambion, Thermo Fisher, USA) were used for the purification of poly(A)‐mRNA according to the manufacturer's instructions. The mRNA was fragmented to ~150 nucleotides in length using the NEB‐Next® Magnesium RNA Fragmentation Module, the fragmented RNA was then incubated with m^6^A antibody (202003, Synaptic Systems, Germany) at 4 °C for 2 h, and then the IP product was used for cDNA synthesis and then selected by AmPure XP beads, the length of the final library is 300 ± 50 bp. Then Illumina Novaseq™ 6000 was used for 2 × 150 bp paired‐end sequencing (PE150). The data obtained after sequencing was filtered, and the clean reads were mapped with the pig (*Sus scrofa*) reference genome *Sscrofa10*.*2*, and the distribution of sequencing sequences was counted. The MACS2.0 software was used to analyse the enrichment distribution of the m^6^A peak in the genome transcripts and different functional regions.

### 4.5 Production of plasmid and lentiviral vector infection

The lentiviral of METTL14 overexpression (METTL14‐OE) was generated by subcloning the CDS of porcine METTL14 into pHBLV‐CMV‐MCS‐EF1‐ZsGreen lentiviral vectors (HanBio, Shanghai, China). The CDS of METTL14 Lentiviral short‐hairpin RNA constructs for porcine METTL14 (M14 KD) were obtained by annealing siRNA oligo sequences and inserted into pHBLV‐U6‐MCS‐CMV‐ZsGreen lentiviral vectors (HanBio, Shanghai, China). For the SP1 overexpression plasmid (pcDNA3.1(+)‐SP1), the CDS of porcine SP1 was inserted into pcDNA3.1 (+) vector (Invitrogen, Cashman, CA, USA) followed by a Flag sequence between *XhoI* and *HindIII* sites. The primer sequences are listed in Table S1.

### 4.6 ChIP assay

ChIP assay was performed by using the ChIP Assay Kit (17–295, Millipore) according to the instructions. Briefly, 5 × 10^6^ pBMSCs were crosslinked with 1% formaldehyde for 10 min and quenched by 200 mM glycine, crosslinked cells were re‐suspended with lysis buffer containing proteinase inhibitors (Sigma) and subjected to sonicate chromatin into 200–1000 bp fragments by a Sonicator (SONICS, VCX 130). Then samples were immunoprecipitation (IP) with 5 μg anti‐SP1 antibody (Abcam, ab13370) at 4°C overnight. The antibody‐bind chromatin was then reversed by incubating with 0.1% SDS and 1 mg/mL Proteinase K (Sigma) at 65°C for 4 h. The ChIP DNA was extracted by using ChIP‐DNA clean and a concentrator kit (Zymo research) and then using for semi‐PCR and qPCR analysis. The primer sequence for ChIP‐PCR is listed in Tables S1.

### 4.7 DNA extraction and biBSP

Genomic DNA was extracted using a TIANamp Genomic DNA Kit (TIANGEN, Beijing, China) in accordance with the provided instructions. After quantifying the concentration, genomic DNA was subjected to bisulphite conversion and then followed by PCR using BSP primers (http://www.urogene.org/methprimer; Tables S1). PCR products were inserted into T‐vector (Takara, Dalian, China), and 10 positive clones were subsequently sent for sanger sequencing (Comate Bioscience, Changchun, China). BioXM (Version 2.6) and MSRcall software were used for the alignment of promoter sequences with the reference genome and for DNA methylation visualization.

### 4.8 Luciferase activity assays

The promoter of METTL14 with different lengths (−2000, −1518, −1000, and −558 bp) was inserted into the PGL4.10‐basic vector (Promega) between *XhoI* and *BGLII* sites. The primer sequences for the METTL14 promoter were given in Table S1. The mutated vectors of the METTL14 promoter were generated by a Q5® Site‐Directed Mutagenesis Kit (E0552S, NEB) in which two SP1 binding sites were mutated from 5′‐AAGAGGCGGGAC‐3′ and 5′‐CCGCCGCTGG‐3′ to 5′‐TTAATTTCAAAGC‐3′.

For in vitro promoter methylated assays, METTL14 promoter without methylated CpG was incubated with CpG methyltransferase *M*.*SssI* (M0226S, NEB) and SAM (B9003S, NEB) at 37°C for 1 h, then *M*.*SssI* was deactivate at 65°C for 20 min. METTL14 promoter with or without *M*.*SssI* treated were transfected into cells and then lysed for luciferase activity detection.

For the quantification of specific RNA m^6^A sites, the WT and Mut TOP2B mRNA sequence contained WT or Mut motifs (where m^6^A replaced by T) were synthesized and inserted into pmirGLO vector (Promega) with *XhoI* and *XbaI* sites. Luciferase activity was evaluated utilizing the Dual‐luciferase Reporter Assay System (Promega, E1910).

### 4.9 RNA decay assay

SCNT embryos were collected after incubating with 5 mg/mL Actinomycin D for 1, 2, 3, and 4 h, then total RNA was isolated, and mRNA was quantities by qRT‐PCR assay. The RNA degradation rate (*k*) was calculated as follows:

$$\ln\left(N/N_{0}\right)=-K_{\text{decay}}t$$

Here, ‘*t*’ represents the duration of transcription inhibition, while ‘*N*’ signifies the mRNA abundance at the specific time point ‘*t*’. ‘*N*_0_’ corresponds to the mRNA level at the initiation of the experiment (0 h), signifying the mRNA content prior to the commencement of decay. Therefore, the mRNA half‐life (*t*_1/2_) can be determined by calculating the degradation rate using the following equation:

$$\text{In}\left(1/2\right)=-K_{\text{decay}}t_{1/2.}$$

### 4.10 scRNA‐seq and bioinformatics analysis

Gene expression levels in embryos were quantified using the Smart‐Seq2 method, following the established protocol as previously described.^37^, ^74^ Briefly, zona pellucida‐free embryos were lysed with lysis buffer (supplemented with ERCC spike‐in). Then samples were subjected to get ṅ through reverse transcription and template switching. After SMART amplification, the Ampure XP beads were used for the purification of cDNA and the concentration was measured by Qubit 2.0 (Thermo Fisher Scientific, MA, USA) was used for measuring the concentration of the DNA. An Agilent 2100 Bioanalyzer was used to assess the size and quality of the insert fragments. Subsequently, the libraries were sequenced by Illumina platform (PE150). Clean reads data were mapped on to *Sus_scrofa 11*.*1*. Transcripts per million was used for the quantification of gene expression levels. Functional enrichment analysis was conducted using DEGs with the threshold of FoldChange >2 and *Q*‐value <0.05.

### 4.11 Quantitative analysis of m^6^A level in total RNA

Total RNA (~200 ng) was isolated from donor cells or embryos. RNA m^6^A level was quantified by an EpiQuik m^6^A RNA Methylation Quantification Kit (P‐9005, Epigentek) following the manufacturer's instruction.

### 4.12 IF staining

Cells with different treatments were washed with PBS three times and then fixed with paraformaldehyde (4%) for 30 min at room temperature. Then the fixed cells were treated with 0.2% Triton X‐100 in PBS for a duration of 10 min. For oocytes or embryos IF staining, acid Tyrode's acid solution (pH 2.5) was used to the remove zona pellucida of oocytes and embryos. After washing with PBS/polyvinylpyrrolidone (PVP) solution, oocytes and embryos were fixed and permeabilized with the same condition as cells. Cells or embryos were blocked with 4% bovine serum albumin (BSA) (w/v) in PBS for 10 min. the cells were then incubated with primary antibodies overnight at a temperature of 4°C. Subsequently, cells or embryos were washed with PBS/PVP three times and stained with corresponding secondary antibodies for 2 h at 37°C. For the nuclei staining, cells or embryos were incubated with 4',6‐diamidino‐2‐phenylindole (DAPI) (10 μg/mL) or Hoechst solution (20 mM) for 10 min. The antibody information has been listed in Table S2.

### 4.13 Microinjection of siRNA

siRNA for TOP2B was designed and synthesized by GenePharma (Shanghai, China). For microinjection, after incubating with PZM3 medium for 4 h, 5–10 pL siRNA or control siRNA were injected into reconstructed embryos by using a microinjection meter (Eppendorf, USA) (Parameter: 220 hpa, 0.8 s), then embryos were transferred into PZM3 medium and cultured at 38.5°C, 5% CO_2_ and 95% air for 7 days.

### 4.14 RNA isolation and qRT‐PCR

For cells, total RNA was isolated from 1 × 10^7^ cells by AllPrep® DNA/RNA Micro Kit (Qiagen, Shanghai, China) according to the manufacturer's instructions. cDNA was synthesized with Transcript All‐in‐one First‐Strand cDNA Kit (TransGen, China). For embryo samples, total RNA was extracted from 200 blastomeres and complementary DNA (cDNA) was synthesized using the SuperScript™ IV CellsDirect™ cDNA Synthesis kit (Invitrogen, USA). This process adhered to the instructions provided by the manufacturer. StepOnePlus™ Real‐Time PCR system (Applied Biosystems) was used for qPCR assay. The primer sequences for qPCR are listed in Table S1. The mRNA level was analysed by the 2^−ΔΔCt^ method.

### 4.15 Protein extraction and western blot

Protein extraction and immunoprecipitation of cells and embryos were prepared as previously described.^34^ Briefly, cells or embryos were lysed by RIPA buffer with a proteinase inhibitor. The lysates were separated by sodium dodecyl‐sulfate polyacrylamide gels and transferred to a polyvinylidene difluoride membrane in the transfer buffer. The blots were blocked with 5% non‐fat milk in 0.1% Tween 20 detergent and then incubated with primary antibodies (Tables S2) at 4°C overnight, and then was incubated with secondary antibodies at 1:5000 dilution.

### 4.16 SELECT assay for the detection of RNA m^6^A site

The Single‐base Elongation and Ligation‐based qPCR Amplification method, abbreviated as ‘SELECT’, was employed to measure RNA m^6^A levels at distinct RNA m^6^A sites.^75^ Briefly, 17 μL 1× CutSmart buffer, 40 nM upstream primer and downstream primer, and 5 μM deoxynucleotide triphosphate were mixed and then incubated with the following procedure: every temperature (90, 80, 70, 60, 50, and 40°C) for 1 min. Subsequently, added Bst 2.0 DNA polymerase (0.01 U) (M0537l, NEB, USA), Splint ligase (0.5 U) (M0375l, NEB, USA), and ATP (10 nM) (P0756S, NEB, USA) with a total volume of 3 μL. The reaction mixture was incubated at 40°C for 20 min, 80°C for 20 min, and then used for qPCR. The primer sequences for the SELECT assay were listed in Table S1.

### 4.17 TUNEL assay

Embryos with zona pellucida removed were washed with 1% PBS/BSA solution for three time, permeabilized by 1% Triton‐X 100/PBS, and then incubated with TUNEL solution (Roche, Mannheim, Germany) for 1 h at 37°C. Embryos were staining with DAPI for 15 min after washing with 1% PBS/Polyvinyl alcohol (PVA) for three times. Subsequently, it was examined using a fluorescence microscope (Nikon, Tokyo, Japan). To control for experimental fluctuations, we kept the same parameters consistent across all replications.

### 4.18 ChIP‐seq and ATAC‐seq data processing

The SP1 ChIP‐seq for human K562 cells (ENCSR341QCG) was obtained from Encode database, the SP1 ChIP‐seq for mouse B cells (GSE164906^75^) and ATAC‐seq data (GSE173275^41^) for porcine pEFs and iPSCs were obtained from GEO database. Clean reads were mapped to the reference genome (GRCh38 for human and mm10 for mouse) with bowtie2, and call peak by MACS2 software with the threshed of *p*‐value <0.001 and >150 bp.

### 4.19 Statistical analysis

All experiments were performed at least three times. The data were presented as the mean ± standard error. Statistical analysis was conducted using GraphPad software, utilizing one‐way analysis of variance (ANOVA) for single‐factor comparisons and two‐way ANOVA when applicable. Additionally, *t*‐test analyses were employed as appropriate for specific comparisons. *p < 0.05 indicated a significant difference, **p < 0.01 indicated a very significant difference.

## AUTHOR CONTRIBUTIONS

Ziyi Li conceived and supervised the project. Meng Zhang mainly performed the experiments and bioinformatics analysis. Yanhui Zhai and Xinglan An helped constructed plasmid and collected the samples. Qi Li, Daoyu Zhang, Yongfeng Zhou, and Sheng Zhang participated in part of the experiment. M. Zhang wrote the article with the help of all the authors. Ziyi Li and Xiangpeng Dai revised the article.

## FUNDING INFORMATION

This project was supported by the National Key R&D Program of China (No. 2017YFA0104400), the Program for Changjiang Scholars, Innovative Research Team in University (No. IRT_16R32), and the National Natural Science Foundation of China (No. 31972874).

## CONFLICT OF INTEREST STATEMENT

The authors declare no competing interests.

## CONSENT FOR PUBLICATION

All co‐authors have read and approved of its submission to this journal.

## Supporting information


**TABLE S1.** Primer sequences used in this study.
**TABLE S2.** Antibody information.
**FIGURE S1.** CpG island of METTL14 promoter region.
**FIGURE S2.** SP1 binding site on METTL14 in human and mouse.

## Data Availability

The data that support the findings of this study are available from the corresponding author upon reasonable request. Sequencing data were deposited into GSA database with accession numbers PRJCA008424 and PRJCA015490.
