# Supplementary material for: DNA methylation regulates RNA m6A modification through transcription factor SP1 during the development of porcine somatic cell nuclear transfer embryos
Source: Cell Prolif. 2023 Dec 14;57(5):e13581. doi: 10.1111/cpr.13581 (PMC11056710; doi:10.1111/cpr.13581)
Supplement: Supplementary file 1 — TABLE S1. Primer sequences used in this study. TABLE S2. Antibody information. FIGURE S1. CpG island of METTL14 promoter region. FIGURE S2. SP1 binding site on METTL14 in human and mouse. [file CPR-57-e13581-s001.pdf]

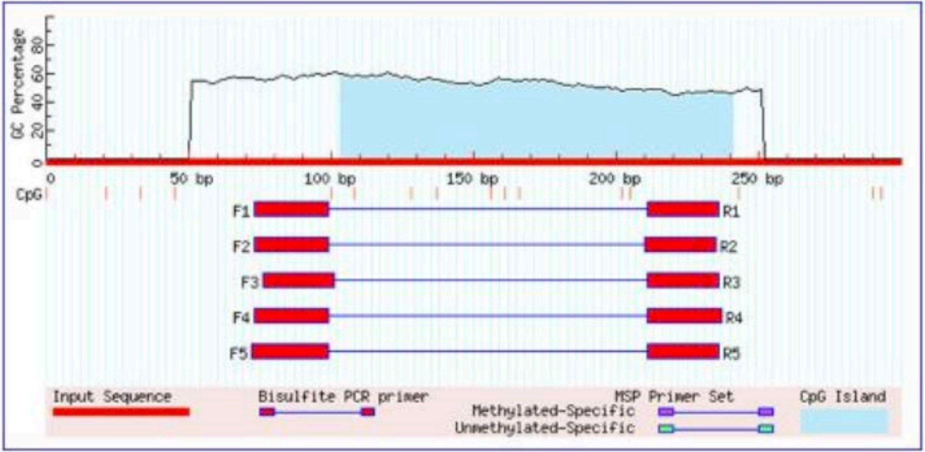
GC Percentage
0
20
40
60
80
0
50 bp
100 bp
150 bp
200 bp
250 bp
CpG
F1
R1
F2
R2
F3
R3
F4
R4
F5
R5
Input Sequence
Bisulfite PCR primer
MSP Primer Set
Methylated-Specific
Unmethylated-Specific
CpG Island

Human

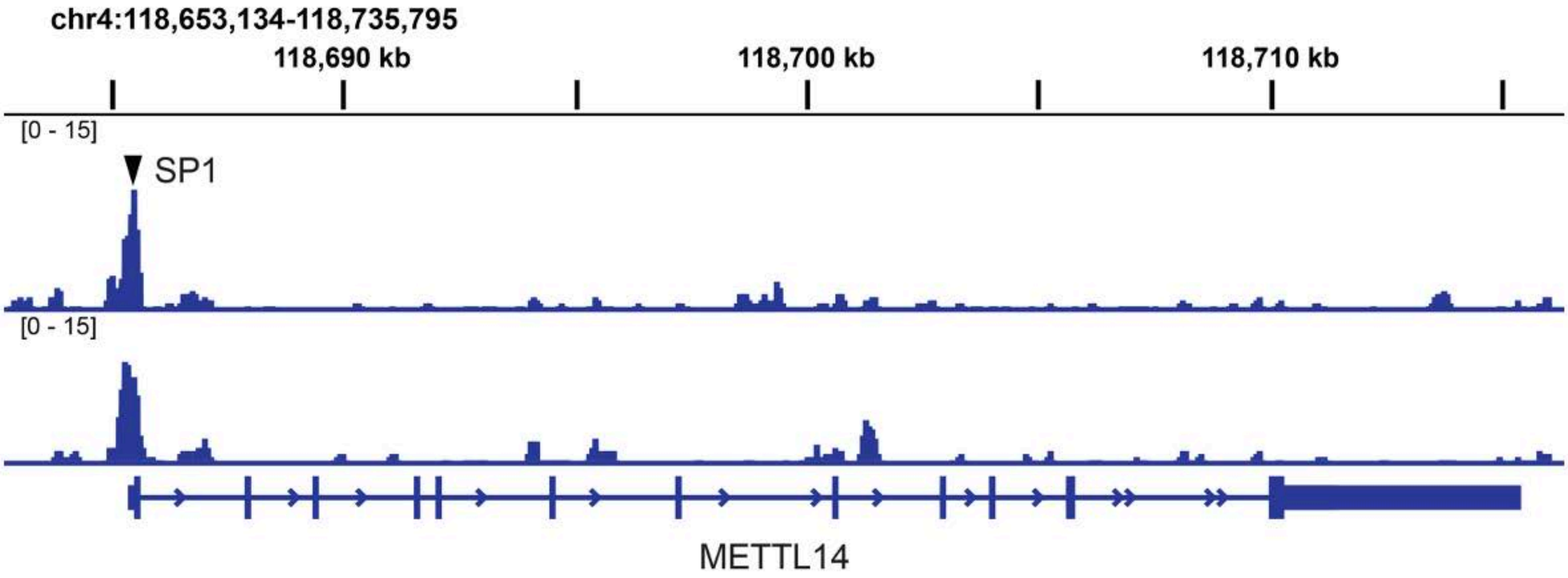
chr4:118,653,134-118,735,795
118,690 kb
118,700 kb
118,710 kb
[0 - 15]
SP1
[0 - 15]
METTL14

Mouse

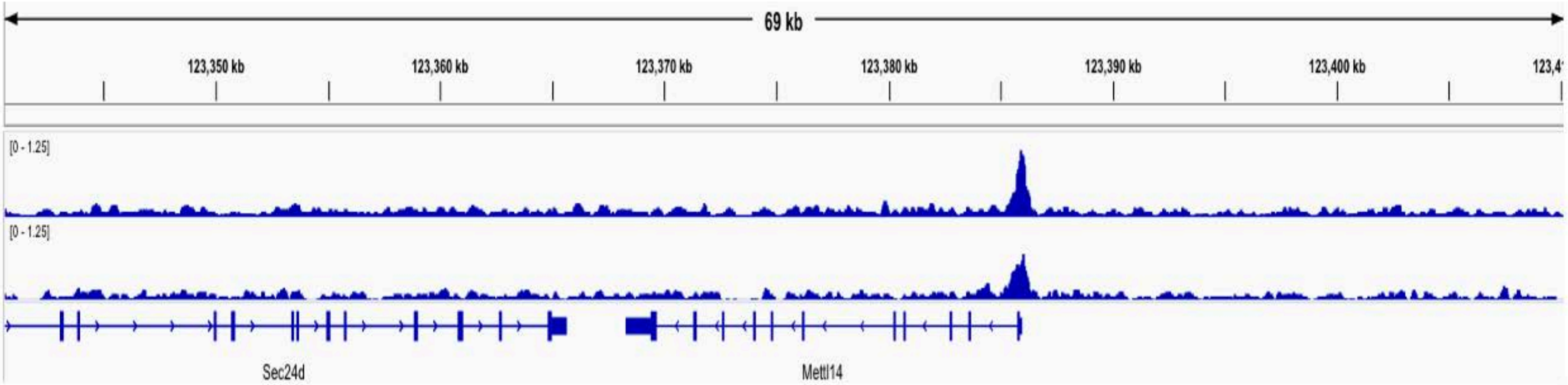
69 kb
123,350 kb
123,360 kb
123,370 kb
123,380 kb
123,390 kb
123,400 kb
[0 - 1.25]
[0 - 1.25]
Sec24d
Mettl14

Table S1 Primer sequences used in the present study

Gene	Forward primer (5'-3')
METTL3	CTACTCTTGTGACCTATGCTGAT
METTL14	TTGACATCAGAGAACTAACACC
WTAP	TGGCTCAGTACCAACAGCAG
ALKBH5	TGTCGGCTTGGCAAGAAAGA
FTO	GTGCGACCCATGCTGTGCTT
YTHDF1	TCGGAGCAAACAGTGAGGAC
YTHDF2	CCTCCATTGGCTTCTCCTATTC
TOP2B	AACAAAAGGTGGACGGCATG
GAPDH	CAAATTCATTGTCGTACCAG
NCBP2	AGCGTGTGGGTTTTGCTTTG
SP1	CTGCCCCTACTGTAAAGATA
WEE2	AGCGGACATATTTGCCTTGG
BTG4	TGCTCGCATTCATCCTTTGC
MAGI3	GATCGATCACTCAGCCCCAG
BCL-2	CTTACCTGAATGACCACCTAGAGC
BAX	CGGGACACGGAGGAGGTTT
POU5F1	GTCGCCAGAAGGGCAAAC
NANOG	CCCCGAAGCATCCATTTCC
SOX2	CCCTGCAGTACAACTCCATGAC
BSP-M14	GGATTTTTATTGGTTTTTTTGTAGGT
METTL14 OE	CCGGTGAATTCGCCACCATGGACTACA
METTL14 shRNA	GATCCGGGAATCAAAGGAACTGTTTTC
METTL14 P1	GCTCGCTAGC**CTCGAG**ACCTTCATCCT
METTL14 P2	GCTCGCTAGC**CTCGAG**GACTTGGGTCT
METTL14 P3	GCTCGCTAGC**CTCGAG**AAGTCCCTTAA
METTL14 P4	GCTCGCTAGC**CTCGAG**AAGTCCCTTAA
METTL14 P1 Mut1	caaagcTTCCGGTCTGGAGGAGC
METTL14 P1 Mut2	caaagcAAGCTGCTGGAGAATCTTG
METTL14 ChIP	CAAACCGCAAAGCTTTCCGA
SP1-CDS	CCC**AAGCTT**GCCACCATGAGCGACCAA
siRNA control	UUCUCCGAACGUGUCACGUTT
TOP2B siRNA	GAACAAAGCUGGUGUGUCATT
Site1	tagccagtaccgtagtgcgtgTGTTGTTACTTCC
Site2	tagccagtaccgtagtgcgtgTCCAGCATTCGTC
Site3-F	tagccagtaccgtagtgcgtgCTTGCGCTAGTTT

Site4	5phos/CATTTTCACCACAAATTTCAcaga
Site4N	tagccagtaccgtagtgcgtgAACTTTGTGCAGT
qPCR	ATGCAGCGACTCAGCCTCTG

Reverse primer (5'-3')	roduct lengt	Applicatior
GCTCCTTGGCTACTTCTGATG	201bp	qPCR
CCAGAACCACACCAGAGAA	184bp	qPCR
TGGCTCAGTACCAACAGCAG	106bp	qPCR
AGACCACTGTGCTGCCATTA	316bp	qPCR
CGGGACTGGCACCTGGCAT	143bp	qPCR
CACCGACCATGACACACAGA	226bp	qPCR
CTCTCCGTTGCTCAGTTGTC	105bp	qPCR
AAAGCTTTTGGGCTGCAGAG	208bp	qPCR
ACACTCACTCTTCTACCTTTG	90bp	qPCR
AATGATGCGGTCATCCAGAC	97bp	qPCR
GCCTCTGTAACTCATCCGA	212bp	qPCR
AGGAATGTTCGGAAGTTGCC	115bp	qPCR
AACGCAGCCACGATTAACAG	125bp	qPCR
GCTTGACTGGTCTTTGCTGC	134bp	qPCR
CCGACTGAAGAGCGAACCC	182bp	qPCR
CGAGTCGTATCGTCGGTTG	189bp	qPCR
CAGGGTGGTGAAGTGAGGG	125bp	qPCR
CGAGGGTCTCAGCAGATGACAT	101bp	qPCR
GGTGCCCTGCTGCGAGTA	86bp	qPCR
AAAACACAAAA TTCTCCAACAACTT	164 bp	BSP
GATGGATCCCTATCGAGGTGGAAAGCC	1371bp	Cloning
AATTCAAAAAAGGGAATCAAAGGAACT	-	Cloning
CGCCGAGGCC**AGATCT**GGCCCAACTC	2000bp	Cloning
CGCCGAGGCC**AGATCT**GGCCCAACTC	1518bp	Cloning
CGCCGAGGCC**AGATCT**GGCCCAACTC	1000bp	Cloning
CGCCGAGGCC**AGATCT**GGCCCAACTC	558bp	Cloning
aaattaaCCTGGATGCCTGCGATTTG	558bp	Cloning
aaattaaGTACCCAATATTCTTTTCCTC	558bp	Cloning
AATTTGCTCCTCCAGACCGG	128bp	ChIP PCR
CCG**CTCGAG**TCACTTATCGTCGTCATC	2361bp	Cloning
ACGUGACACGUUCGGAGAATT	-	siRNA
UGACACACCAGCUUUGUUCTT	-	siRNA
5phos/TCTGAGCCAAATTCACAATAGcag	-	SELECT
5phos/TCACAATTTCCCTAGCATCATcag	-	SELECT
5phos/CATTTTCACCACAAATTTCAcaga	-	SELECT

5phos/CCAGCAGCTTCTGCTTGCGCcag[illegible]	-	SELECT
5phos/TCTGCTTGCGCTAGTTTCTCcaga	-	SELECT
TAGCCAGTACCGTAGTGCGTG	-	SELECT

ו

Table S2 Antibody information

Antibody	Item No.	Dilution
METTL3	Proteintech, 15073-1-AP	1:1000
METTL14	Sigma-Aldrich, HPA038002	1:1000
ALKBH5	Proteintech, 16837-1-AP	1:1000
FTO	SANTA CRUZ, sc-271713	1:1000
YTHDF1	Proteintech, 17479-1-AP	1:1000
YTHDF2	Proteintech, 24744-1-AP	1:1000
GAPDH	Proteintech, 60004-1-Ig	1:3000
RNA m6A	SYSY, 202003	1:100
γH2AX	Cell signaling, 2577	1:200
Secondary Antibody	Invitrogen, A-11001	1:500
Secondary Antibody	Invitrogen, A-11037	1:500

esource specie	pplication
Rabbit	WB
Rabbit	WB
Rabbit	WB
Rabbit	WB
Rabbit	WB
Rabbit	WB
Rabbit	WB
Rabbit	IF
Rabbit	IF
Goat anti-Mouse	IF
Goat anti-Rabbit	IF
